# Novel oxygen sensing mechanism in the spinal cord involved in cardiorespiratory responses to hypoxia

**DOI:** 10.1126/sciadv.abm1444

**Published:** 2022-03-25

**Authors:** Nicole O. Barioni, Fatemeh Derakhshan, Luana Tenorio Lopes, Hiroshi Onimaru, Arijit Roy, Fiona McDonald, Erika Scheibli, Mufaddal I. Baghdadwala, Negar Heidari, Manisha Bharadia, Keiko Ikeda, Itaru Yazawa, Yasumasa Okada, Michael B. Harris, Mathias Dutschmann, Richard J. A. Wilson

**Affiliations:** 1 Department of Physiology and Pharmacology, Hotchkiss Brain Institute and Alberta Children’s Hospital Research Institute, Cumming School of Medicine, University of Calgary, Calgary, Alberta, Canada.; 2 Department of Physiology, Showa University School of Medicine, Tokyo, Japan.; 3 Division of Internal Medicine, Murayama Medical Center, Musashimurayama, Tokyo, Japan.; 4 Global Research Center for Innovative Life Science, Peptide Drug Innovation, Hoshi University School of Pharmacy and Pharmaceutical Sciences, Tokyo 142-8501, Japan.; 5 Department of Biological Sciences, California State University Long Beach, Long Beach, CA 90840, USA.; 6 Florey Institute of Neuroscience and Mental Health, University of Melbourne, Melbourne, Victoria, 3052, Australia.

## Abstract

As blood oxygenation decreases (hypoxemia), mammals mount cardiorespiratory responses, increasing oxygen to vital organs. The carotid bodies are the primary oxygen chemoreceptors for breathing, but sympathetic-mediated cardiovascular responses to hypoxia persist in their absence, suggesting additional high-fidelity oxygen sensors. We show that spinal thoracic sympathetic preganglionic neurons are excited by hypoxia and silenced by hyperoxia, independent of surrounding astrocytes. These spinal oxygen sensors (SOS) enhance sympatho-respiratory activity induced by CNS asphyxia-like stimuli, suggesting they bestow a life-or-death advantage. Our data suggest the SOS use a mechanism involving neuronal nitric oxide synthase 1 (NOS1) and nicotinamide adenine dinucleotide phosphate (NADPH) oxidase (NOX). We propose NOS1 serves as an oxygen-dependent sink for NADPH in hyperoxia. In hypoxia, NADPH catabolism by NOS1 decreases, increasing availability of NADPH to NOX and launching reactive oxygen species–dependent processes, including transient receptor potential channel activation. Equipped with this mechanism, SOS are likely broadly important for physiological regulation in chronic disease, spinal cord injury, and cardiorespiratory crisis.

## Teaser

Spinal oxygen sensors use a novel O_2_ sensing mechanism to increase breathing and sympathetic activity with onset of asphyxia.

## INTRODUCTION

In mammals, carotid bodies are the primary oxygen sensors driving breathing, sympathetic activity, and blood pressure in response to acute hypoxia (*1*, *2*). While carotid body denervation abolishes most but not all of the hypoxic ventilatory response (*3*), some of the sympathetic responses—and, consequently, cardiovascular effects—remain (*4*, *5*). Thus, unlike brainstem neuronal networks controlling breathing, mostly inhibited by acute hypoxia in the absence of the carotid bodies, sympathetic networks must be strongly excited (*6*, *7*). However, the molecular and cellular identity of the central sympathetic oxygen sensor is largely unresolved.

Some studies have identified hypoxia-excited cells in the hypothalamus (*8*) and the brainstem, including the nucleus tractus solitarius (NTS) (*9*) and rostral ventrolateral medulla (RVLM) (*7*, *10*). These cells receive carotid body inputs and activate brainstem respiratory and/or spinal thoracic preganglionic sympathetic neurons (*11*). The existence of central oxygen sensors may explain reports of proexcitatory effects of brainstem hypoxia under some conditions (*3*, *12*). While mechanisms of oxygen sensing are not fully resolved, they likely involve astrocytes, heme oxygenase (HO), Na_p_ channels, and/or adenosine 5′-monophosphate–activated protein kinase (*13*–*15*, *26*). However, the existence of oxygen sensors in the brainstem and hypothalamus do not exclude the possibility that the spinal cord is also oxygen sensitive.

Indeed, data from 1909 using spinalized animals point to oxygen-sensitive spinal “vasomotor centers” (*16*). However, a multitude of potential mechanisms may be at play in vivo, causing controversy as to the ability of the spinal cord to mediate the carotid body–independent pressor effects of hypoxia (*17*–*19*). For example, in vivo spinalized preparations do not eliminate the possibility that spinal circuits are activated by brainstem and systemic effects of hypoxia, such as release of lactic acid or hormones (*20*). Consequently, while recent interest has surged in brainstem oxygen sensing, with astrocyte activation proposed as a mechanism to offset central hypoxic inhibition (*21*–*23*), the possibility of powerful cardiovascular spinal oxygen sensors (SOS) has been overlooked. Here, we draw on artificially perfused in situ and en bloc preparations to overcome the limitations of previous in vivo studies. We confirm that spinal cord sympathetic preganglionic neurons are oxygen sensitive and describe experiments to determine their sensitivity, critical function, cellular identity, and mechanism.

## RESULTS

**Fig. 1.**
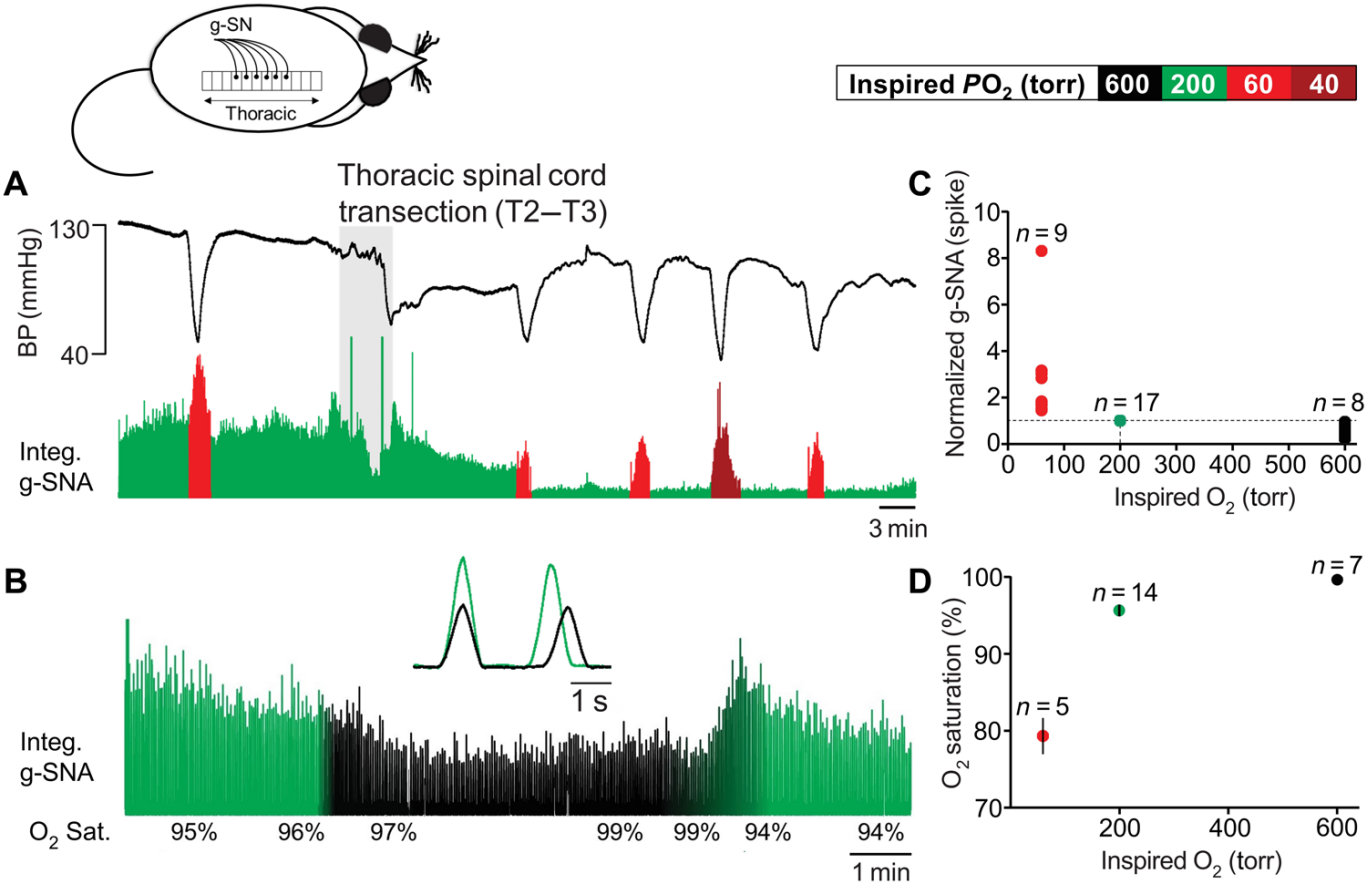
The spinal cord is sensitive to changes in oxygen in vivo. Urethane anesthetized, vagotomized, artificially ventilated, and carotid body and aortic arch–denervated rat in vivo preparation. (**A**) Greater splanchnic nerve activity (g-SNA; rectified) responses to changes in inspired O_2_ (box). Hypoxia increases g-SNA before and after surgical removal of the T2 and T3 spinal cord segment (gray). BP, blood pressure; Integ. g-SNA, integrated and rectified g-SNA. (**B**) After transection, hyperoxia reduced g-SNA, suggesting that the SOSs contribute to sympathetic tone in normoxia. Inlay: Triggered averages of 10 g-SNA burst pairs before (green) and during (black) hyperoxia. (**C** and **D**) Posttransection group data. Hypoxia (red circles; ventilated with 60-torr *P*O_2_; 79.3 ± 2.4 SO_2_) increases g-SNA compared to normoxia (green circles; inspired *P*O_2_ of 200 torr, SO_2_ of 95.6 ± 0.7%; Wilcoxon signed-rank test, *P* = 0.004), while hyperoxic reduces activity (black circles; inspired *P*O_2_ of 600 torr with g-SNA measured at SO_2_ of 99.7 ± 0.1%; Wilcoxon signed-rank test, *P* = 0.008). Note that, in these anesthetized preparations, hypoxic-induced sympathoexcitation is not sufficient to reverse the hypotension caused by the peripheral vasodilatory effects of hypoxia.

To test for the SOS, we recorded greater splanchnic nerve [g-SN; sympathetic thoracic root 5 (T5) to T9] activity in anesthetized, paralyzed, vagotomized, carotid body and aortic arch–denervated and artificially ventilated rat preparations. The g-SN was chosen because of its surgical accessibility and the fact that it is heavily endowed with axons from spinal intermediolateral nucleus (IML) spinal thoracic sympathetic preganglionic neurons (SSPNs) (*24*). Under baseline conditions, preparations were ventilated with gas having a partial pressure of oxygen (*P*O_2_) of 200 torr (balance N_2_). Despite the lack of arterial chemoreceptors, all preparations responded to a bout of hypoxia (1 to 2 min of 60 torr) with increased g-SN activity. Following spinal cord transection at T2 and T3, baseline g-SN activity decreased. However, despite no arterial chemoreceptors or descending inputs from the brainstem, preparations maintained their vigorous sympathetic response to hypoxia (Fig. 1A). Thus, g-SN activity (spike) significantly increased when the gas used to ventilate animals was switched from 200- to 60-torr *P*O_2,_ causing oxygen saturation to decrease to 79.3 ± 2.4% from 95.9 ± 0.5% (change in g-SN activity: Wilcoxon signed-rank test, *P* = 0.004; *n* = 9). In a separate set of spinalized preparations, increasing inspired *P*O_2_ from 200 to 600 torr, causing a change in saturation from 95.4 ± 1.2% to 99.7 ± 0.1% (Wilcoxon matched-pairs signed-rank test, *P* = 0.016; *n* = 7), resulted in a decrease in g-SN activity (Wilcoxon signed-rank test, *P* = 0.008; *n* = 8; Fig. 1B). These results are summarized in Fig. 1 (C and D). These data suggest the spinal cord contains oxygen sensors that contribute to SSPN activity, even when blood oxygen saturation is close to normal. Alternatively, changes in SSPN activity could be produced by blood-borne neural modulators released by the hypothalamus, brainstem, lung, adrenal gland, or some other oxygen-sensitive organs.

**Fig. 2.**
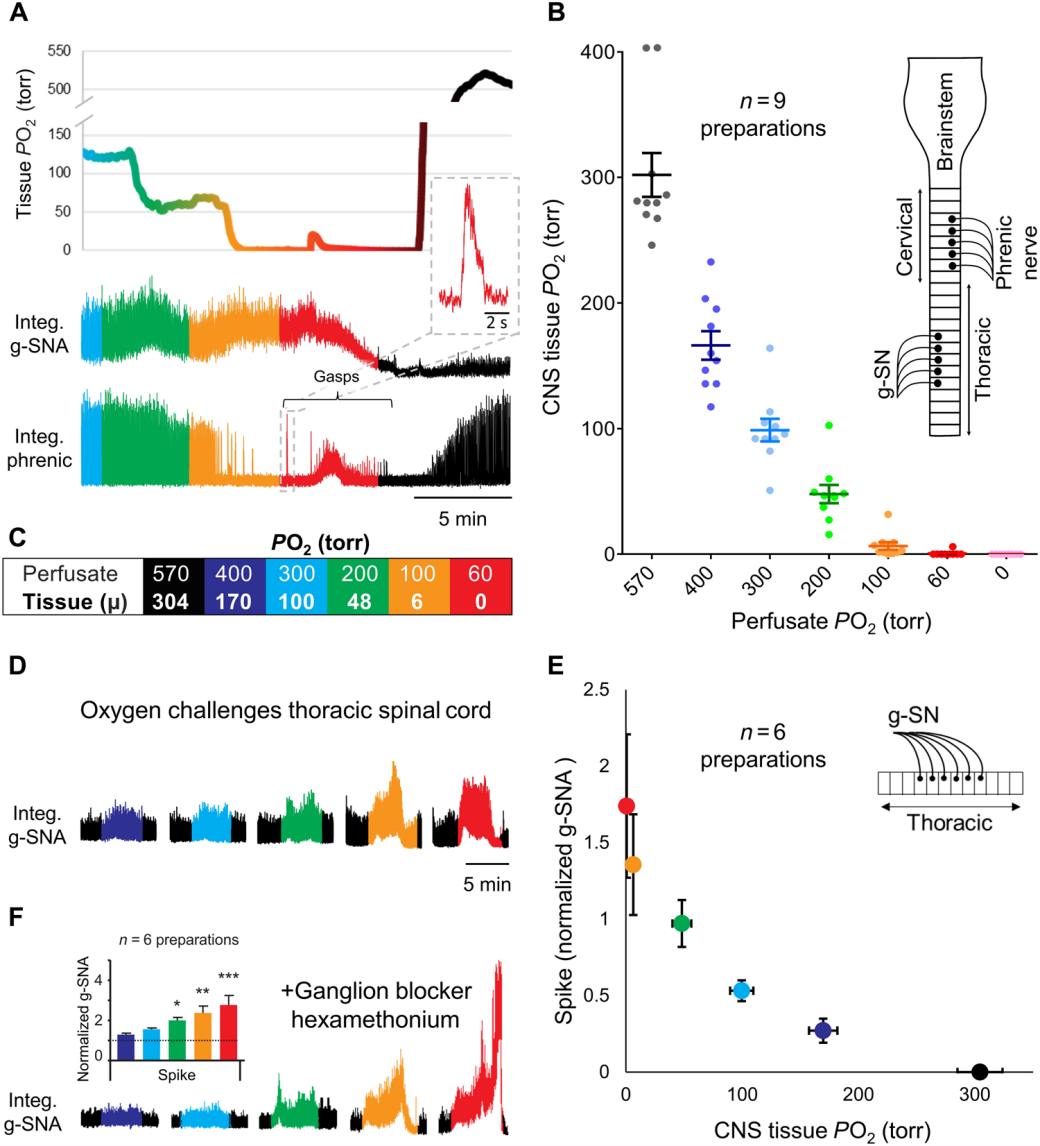
Oxygen sensitivity of the spinal cord is retained in situ. (**A** and **B**) Dual perfused in situ preparation with independent perfusion of the carotid bodies and central nervous system (CNS) (brainstem and spinal cord) compartments [see inlay in (B)], demonstrating relationship between perfusion *P*O_2_ and CNS tissue *P*O_2_. CNS tissue oxygenation was measured in the brainstem with a Clark-style polarographic microelectrode (tip diameter, <25 μm; graph shows means ± SEM), while oxygenation of the central perfusate was gradually decreased from 570- to 60-torr *P*O_2_ (*P*CO_2_ maintained at 40 torr) in 5-min step decrements. Carotid body perfusate was kept constant throughout (35-torr *P*CO_2_ and 100-torr *P*O_2_). Note that the increase in g-SNA as CNS *P*O_2_ enters the physiological range [i.e., 30 to 50 torr (*25*)] and cessation of eupneic phrenic discharge and initiation of gasps when CNS *P*O_2_ becomes severely hypoxic (<10-torr *P*O_2_). (**C**) Color-coded box summarizes the relationship between perfusate *P*O_2_ and mean (μ) tissue *P*O_2_. (**D** and **E**) In situ thoracic spinal cord preparation [see inlay in (E)] demonstrating relationship between g-SNA and tissue *P*O_2_ established in (B) (g-SNA normalized to tissue *P*O_2_ of 50 torr approximating that of in vivo CNS normoxia). For comparison to O_2_ sensitivity of carotid bodies, see fig. S1. (**F**) g-SNA responses persisted in the in situ spinal cord preparation in the presence of the ganglionic blocker hexamethonium (100 μM; table S2), suggesting that oxygen sensitivity is not dependent on synaptic connections within the sympathetic chain. Integ. phrenic, integrated and rectified phrenic nerve activity. Amplitude and/or area under the curve indicates activity level. Bars show means ± SEM; see color key in (C) for tissue *P*O_2_. *P < 0.05, **P < 0.01, and ***P ≤ 0.001.

To definitively demonstrate the existence of SOS, we developed a novel in situ thoracic spinal cord preparation, perfused with artificial cerebrospinal fluid (aCSF) via the descending aorta. Lungs, diaphragm, heart, kidneys, and brainstem were absent. With the ventral side up, sympathetic activity was recorded from the g-SN while changing perfusate *P*O_2_. As expected, g-SN activity increased as perfusate *P*O_2_ was reduced (Fig. 2). Specifically, a reduction in perfusion *P*O_2_ caused a significant increase in g-SN activity (Friedman test, *P* < 0.0001; *n* = 6; for both spike and integral nerve activities). A post hoc analysis found differences between 570-torr and 200-, 100-, and 60-torr *P*O_2_ challenges (Dunn’s multiple comparison test, *P* < 0.05) for both integral and spike nerve activities. When perfusate *P*O_2_ was 200 torr or less, sympathetic activity was more than twice that at a *P*O_2_ of 570 torr.

To determine sensitivity of g-SN responses to changes in *P*O_2_, we attempted to access the spinal cord of the in situ preparation via laminectomy, without disrupting the dorsal vasculature normally supplying a substantial proportion of the oxygenated blood. Despite our best efforts to be minimally invasive, including the use of Clark-style polarographic microelectrode with 25-μm tip diameters, we found that the dorsal surface of the spinal cord at the site of surgery had a *P*O_2_ equal to that of the perfusate. That is, perfusate was flowing over the surface of the tissue from ruptured blood vessels, suggesting the dorsal tissue at the site of the laminectomy was likely under perfused compared to an intact segment. Moreover, without the limitations imposed by the dorsal circulation on inflow from the ventral vasculature, the ventral tissue was likely hyperperfused. Thus, we adopted a different strategy. In contrast to the spinal cord, the brainstem is mostly vascularized from the ventral surface and can be safely accessed from the dorsal side via the fourth ventricle. Exploiting this anatomical difference, we used an artificially perfused brainstem–spinal cord preparation to measured brainstem tissue *P*O_2_ as a surrogate for spinal cord tissue *P*O_2_.

In the in situ brainstem–spinal cord preparation, when the *P*O_2_ of the gas used to equilibrate the perfusate was reduced to 200 torr, tissue *P*O_2_ entered the physiological range [48 ± 24 torr (*25*)], and g-SN activity increased (Fig. 2, A to C). When *P*O_2_ of the equilibrating gas was reduced to 100 torr, the brainstem tissue became hypoxic (6 ± 10–torr *P*O_2_), activity of g-SN was sustained, and phrenic nerve activity became quiescent. A perfusate below 60-torr *P*O_2_ resulted in tissue hypoxia and a transient period of g-SN activity and gasping (rapid onset decrementing phrenic bursts). As the oxygen sensitivity of g-SN recorded in the in situ brainstem–spinal cord preparation is likely influenced by oxygen sensitivity of brainstem circuits, we plotted brainstem tissue *P*O_2_ for a given perfusate *P*O_2_ against g-SN responses obtained in the in situ thoracic spinal cord preparation (Fig. 2, D and E). g-SN activity triggered by low oxygen persisted in the in situ thoracic spinal cord preparation in the presence of ganglionic blocker 100 μM hexamethonium (Friedman test for spike; *n* = 6; see table S2), indicating that synaptic transmission within the sympathetic chain was not necessary for the hypoxic response (Fig. 2F). Together, the data confirm the existence of SOS, suggesting that their sensitivity spans the physiological range and is similar to that of the carotid bodies between 60 and 300 torr (see fig. S1).

**Fig. 3.**
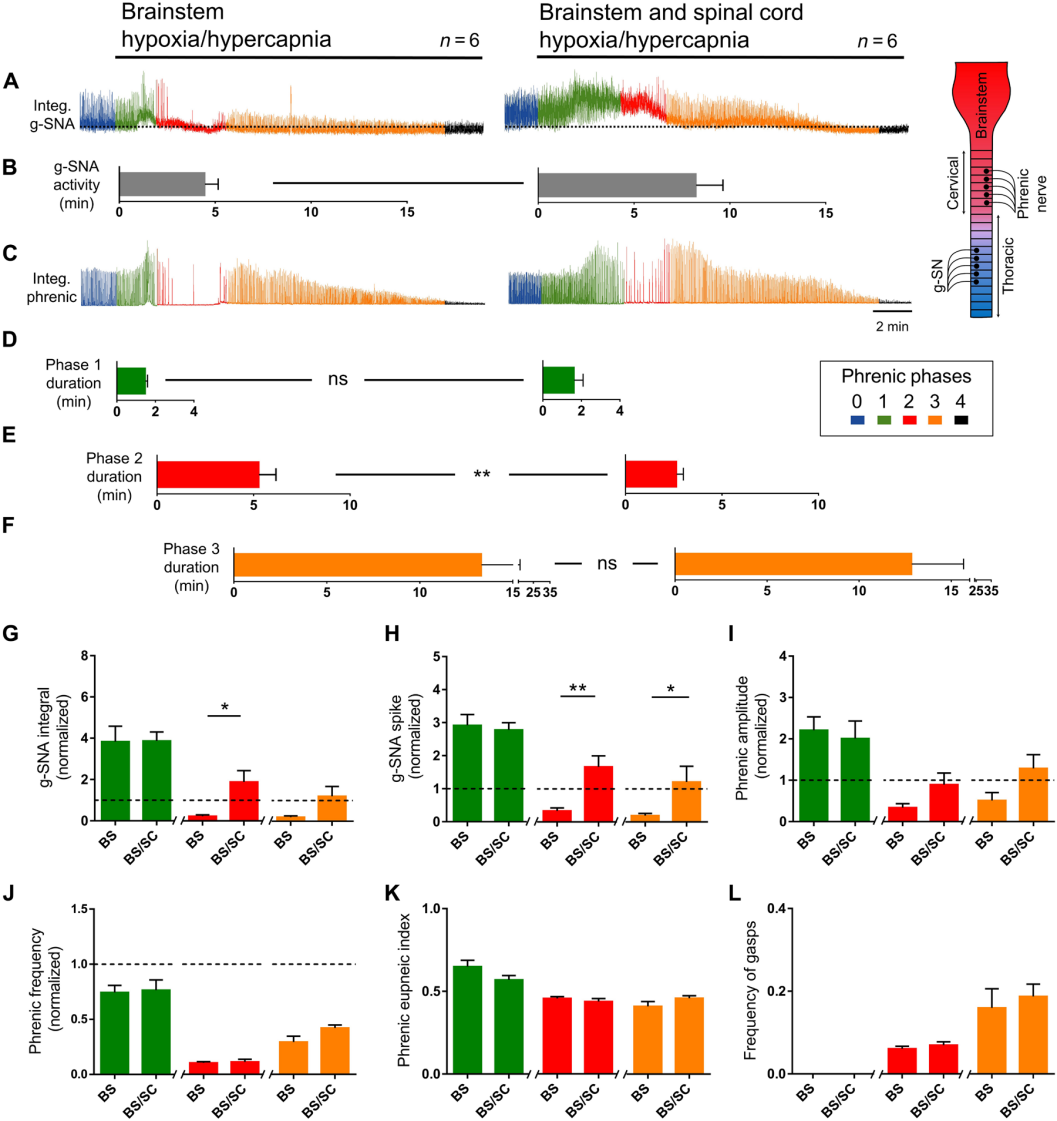
SOS promotes defensive sympatho-respiratory responses to asphyxia-like stimuli. Triple perfused in situ preparation with independent perfusion of carotid bodies, brainstem (+spinal levels C1 to T3) and thoracic spinal cord (T4 to T13) compartments. For verification of brainstem and thoracic spinal cord compartment independence, see fig. S2. Carotid body perfusate was kept constant throughout (35-torr *P*CO_2_ and 100-torr *P*O_2_). Brainstem alone or brainstem plus thoracic compartment were challenged with hypoxic-hypercapnic perfusate (40-torr *P*O_2_/60-torr *P*CO_2_). Respiratory responses divided into five phases based on phrenic nerve activity (box): 0, baseline; 1, hyperpnea; 2, primary apnea and gasp; 3, recovery phase; 4, terminal apnea. (**A** and **B**) Exposing the brainstem compartment alone (left) causes a transient increase in phrenic activity (phase 1), followed by primary apnea and gasps (phase 2). Compared to exposing the brainstem compartment alone, exposing brainstem and thoracic compartments (right) prolongs g-SNA (time to decay to 50% below baseline: unpaired *t* test with Welch’s correction, *P* = 0.045); (**C** to **F**) shortens the apnea/gasp phase (phase 2), promoting faster onset of recovery (phase 3; Mann-Whitney test, *P* = 0.009); (**G** and **H**) increases g-SNA during phases 2 and 3 (unpaired *t* test with Welch’s correction; integral phase 2, *P* = 0.03; spike phase 2, *P* = 0.009; spike phase 3, *P* = 0.04); and (**I** to **L**) has no effect on phrenic burst amplitude, shape, or frequency beyond that of exposing the brainstem alone. Amplitude and/or area under the curve indicates activity level. Bars show means ± SEM. *P < 0.05 and **P < 0.01. ns, not significant; BS, brainstem; SC, spinal cord.

The response to central hypoxia of the in situ brainstem–spinal cord preparation confirms the work of others, indicating that the central nervous system (CNS) is directly involved in mounting cardiorespiratory survival responses. To test whether the SOS themselves contribute to life-saving responses during cardiorespiratory crisis, we developed a new triple perfused in situ preparation in which most of the thoracic spinal cord (T4 to T13), more rostral neuronal structures (rostral thoracic, cervical spinal cord, and brainstem), and carotid bodies are perfused independently (see fig. S2). Respiratory and sympathetic responses to CNS perfusion with an asphyxia-like stimulus [40-torr *P*O_2_ and 60-torr partial pressure of CO_2_ (*P*CO_2_)] with the carotid bodies unchanged were assessed from phrenic and splanchnic nerves, respectively. When only the brainstem compartment was exposed, we observed four phases of activity: (i) increase in amplitude of eupneic-like phrenic bursts and sympathetic activity [demonstrating the significant bulbospinal projections onto SSPNs (*11*, *27*) and the existence of brainstem chemoreceptors (*3*, *10*, *11*)]; (ii) a period of primary apnea and gasp-like phrenic bursts [demonstrating that brainstem asphyxia alone is sufficient to generate gasps (*22*)] during which sympathetic activity gradually rolled off; (iii) augmented phrenic activity with sympathetic activity at/below baseline activity; and (iv) terminal apnea and sympathetic quiescence. In comparison, when both the thoracic and brainstem compartments were exposed to the same stimuli, the period of primary apnea and gasp-like bursts was reduced, shortening the time to augmented phrenic activity (Mann-Whitney test, *P* = 0.009), and the increase in sympathetic activity was prolonged (integral: unpaired *t* test with Welch’s correction, *P* = 0.03; spike: unpaired *t* test with Welch’s correction, *P* = 0.009), persisting until commencements of terminal apnea (Fig. 3 and fig. S3). Thus, inclusion of the SOS in brainstem responses enhances autoresuscitative reflexes and therefore is likely to be prosurvival.

**Fig. 4.**
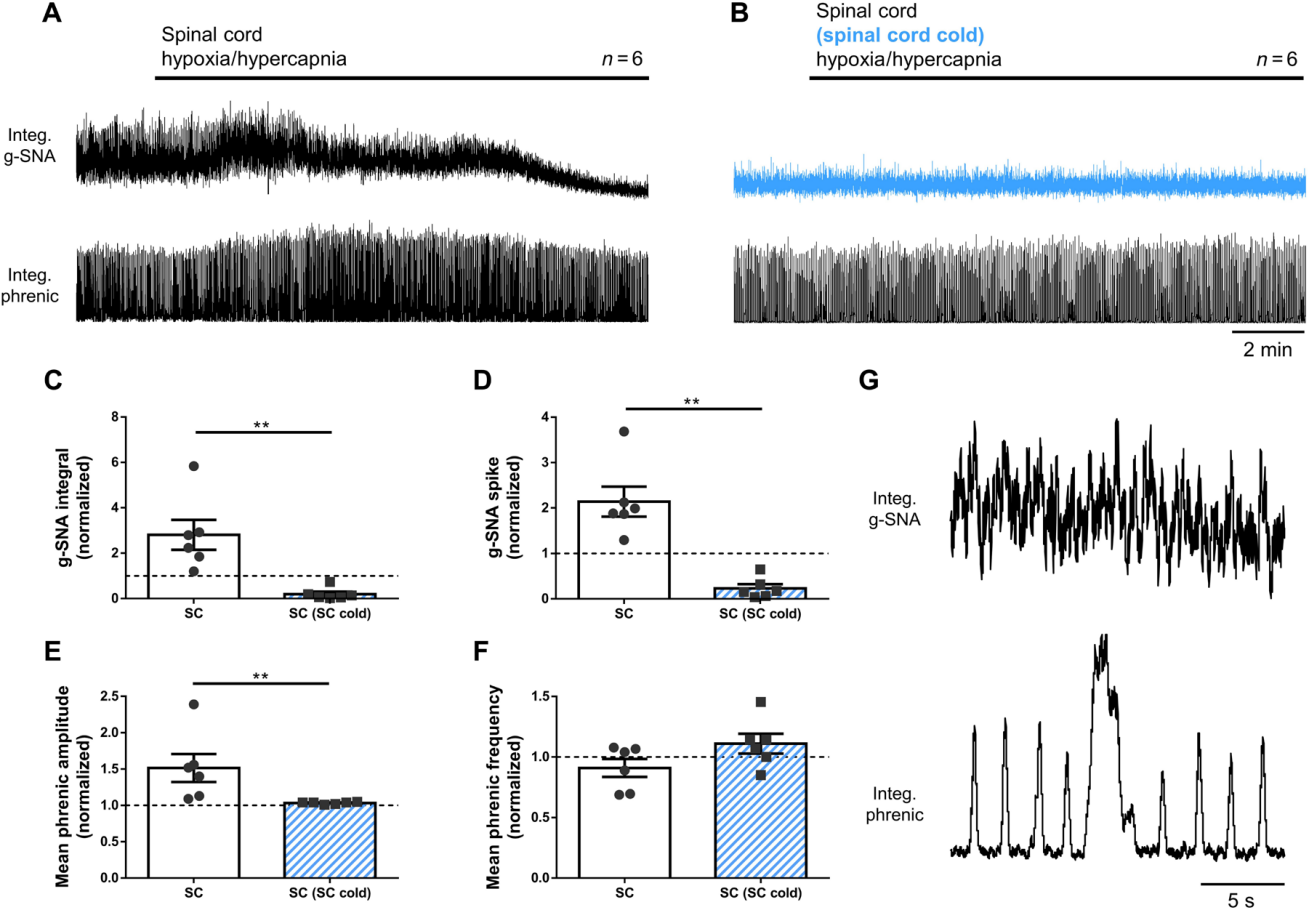
Rostral respiratory and autoresuscitative circuits receive ascending excitatory drive from the SOSs. Triple perfused in situ preparation with independent perfusion of carotid bodies, brainstem (+spinal levels C1 to T3), and thoracic spinal cord (T4 to T13) compartments. (**A**) Challenge of thoracic compartment alone with hypoxic-hypercapnic perfusate (40-torr *P*O_2_, /60-torr *P*CO_2_) causes increase in phrenic and g-SNA. (**B**) Selective cooling (5° to 10°C) of the thoracic segment perfusate (blue) abolishes stimulatory effect of hypoxia/hypercapnia on g-SNA and phrenic activity. (**C** to **F**) Quantified data. (**G**) In three of six preparations, the hypoxic/hypercapnic challenges caused gasps. Amplitude and/or area under the curve indicates activity level. **P < 0.01.

As the spinal cord challenge promotes the prosurvival responses resulting from brainstem challenge, we hypothesize that the SOS provide excitatory drive to autoresuscitative circuits in the brainstem. To test this, we performed additional experiments in which only the spinal cord was challenged (i.e., during the spinal cord challenge, the brainstem was kept hyperoxic and normocapnic). When spinal cord alone was challenged, sympathetic (integral: one sample *t* test versus one, *P* = 0.04; spike: Wilcoxon signed rank test versus one, *P* = 0.031) and phrenic (amplitude: one sample *t* test versus one, *P* = 0.044) activities were again elicited (Fig. 4, A to F). Additionally, three of six preparations produced gasps on a background of continuous eupneic phrenic bursts (Fig. 4G). As the spinal cord is only mildly excited by hypercapnia (fig. S4), these responses appear to be largely triggered by hypoxia, again demonstrating the independent oxygen sensitivity of the thoracic spinal cord. Moreover, these data indicate a caudal-rostral projection from the spinal cord to the phrenic motor nucleus and/or brainstem respiratory centers. Electrophysiological evidence for such a pathway has been described in anesthetized dogs (*28*).

### Identity of oxygen sensing cells

**Fig. 5.**
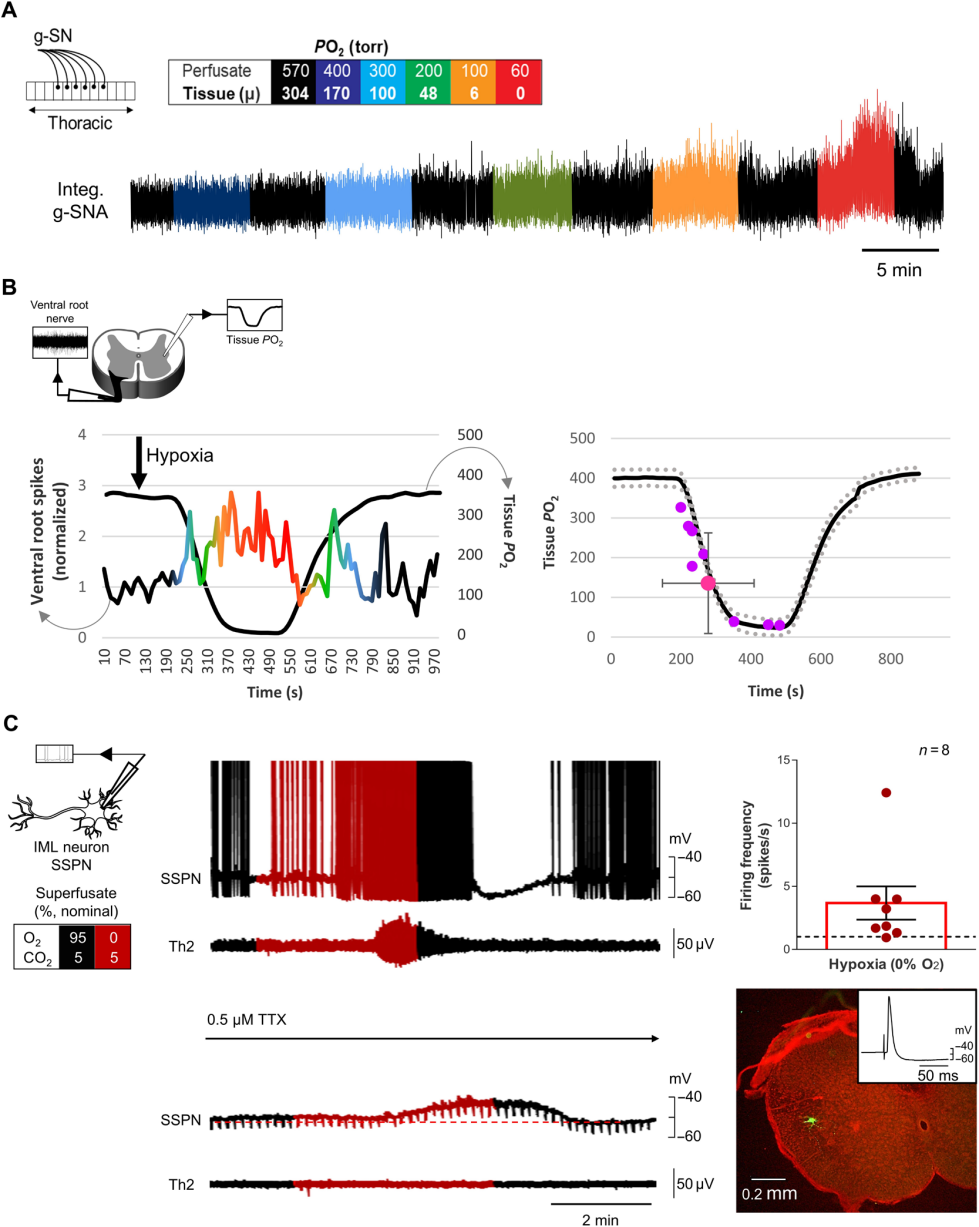
Neonatal sympathetic responses to hypoxia in perfused in situ thoracic spinal cord and en bloc transverse thoracic spinal cord section preparations. (**A**) Recording of neonatal (P2) g-SNA in response to hypoxia in a single perfused thoracic spinal cord in situ preparation (schematic in top left corner). Reducing perfusate *P*O_2_ increases g-SN activity. Box: Color-coding illustrates perfusate *P*O_2_ in torr. Amplitude and/or area under the curve indicates activity level. (**B**) IML tissue *P*O_2_ measurement from Clark-style microelectrode with tip 100 μm below tissue surface with contralateral ventral nerve recording from en bloc transverse thoracic spinal cord section preparation (schematic in top left corner). As tissue *P*O_2_ decreases, ventral nerve activity increases (i.e., mounted responses exceeding four SDs of baseline activity) in 8 of 14 preparations. *P*O_2_ threshold response (as defined above) occurred at 140 ± 121 torr (*n* = 8). (**C**) Individual IML SSPN (whole cell) and ventral root nerve activity (T2) in en bloc preparations. Firing frequency increased (as defined above) in 8 of 11 preparations when aCSF equilibrated with 0% O_2_ was delivered to the dish (giving a dish nadir ~13% FO_2_). During the 1-min peak response, firing rate increased by 3.67 ± 1.32 spikes/s (*n* = 8). In TTX, hypoxia depolarized neurons by 3.1 ± 0.98 mV (repeated-measures one-way ANOVA; baseline versus hypoxia, *P* = 0.03; hypoxia versus washout, *P* = 0.01; *n* = 10) but had no effect on membrane resistance (1049 ± 29 megohms; repeated-measures one-way ANOVA, *P* = 0.5; *n* = 10). Neurons were identified by backfiring the ventral root and filling with Lucifer yellow (illustrated in bottom left panel).

To resolve the identity of the oxygen-sensitive cells, we used a combination of thoracic spinal cord in situ preparations and superfused en bloc transverse thoracic spinal cord sections that necessitated the use of neonates. We first demonstrated that g-SN oxygen sensitivity was present in neonate rats as young as postnatal day 2 (P2) using the in situ preparation (Fig. 5A). Quantification of neonatal oxygen sensitivity was not attempted in the neonatal in situ preparations because of the technical difficult of recording from the tiny g-SN in animals of this age. Instead, we recorded from ventral roots in neonatal en bloc transverse thoracic spinal cord sections from P0 to P4 rats while measuring tissue *P*O_2_ in the contralateral IML 100 μm below the tissue surface. Ventral roots in 8 of 14 en bloc preparations responded to a fall in perfusate *P*O_2_ (defined as activity exceeding four times the SD of baseline activity). Threshold responses occurred at a tissue *P*O_2_ of 140 ± 121 torr (*n* = 8) (Fig. 5B). These responses occurred despite the steep oxygen gradients with depth inherent in all superfused preparations and the loss of intersegmental spinal processes that would be expected to magnify single SSPN responses. We next examined the oxygen sensitivity of individual SSPNs using whole-cell recordings. IML neurons were identified as SSPNs in en bloc preparations from P0 to P4 rats (male and female) by backfiring the ventral root using a suction electrode and intracellular labeling with Lucifer yellow. As expected, soma and ventral root spiking activity increased (as defined above) during hypoxic challenge (dish nadir of ~13% FO_2_) in 8 of 11 preparations (Fig. 5C). In the presence of tetrodotoxin (TTX), SSPN spiking activity induced by hypoxia was abolished in both soma and ventral root. Under these conditions, which functionally isolate each SSPN from inputs from all other spiking neurons, hypoxia (13% O_2_ as measured in dish) caused SSPNs to depolarize by 3.1 ± 1.0 mV and repolarize on return to baseline conditions [87% O_2_ in dish; repeated-measures one-way analysis of variance (ANOVA), *P* = 0.004; *n* = 10]. In 6 of 10 neurons, a biphasic membrane potential response to hypoxic stimulation was observed (e.g., Fig. 5C), such that the hypoxic-induced depolarization was followed by hyperpolarization during reoxygenation. In the absence of TTX, these hypoxic-induced membrane dynamics of individual SSPNs are likely to be substantially amplified by the extensive segmental and intersegmental synaptic interconnections that characterize the intact IML network. Input resistance was unchanged during hypoxic-induced depolarization (repeated-measures one-way ANOVA, *P* = 0.28; *n* = 10), suggesting an important role for release of calcium from internal stores (Figs. 5C; see Effector section below). Detailed statistical analysis of cellular responses is presented in table S1. Together, these data suggest that individual neonatal SSPNs are oxygen sensitive and likely play a role in spinal cord responses to hypoxia.

**Fig. 6.**
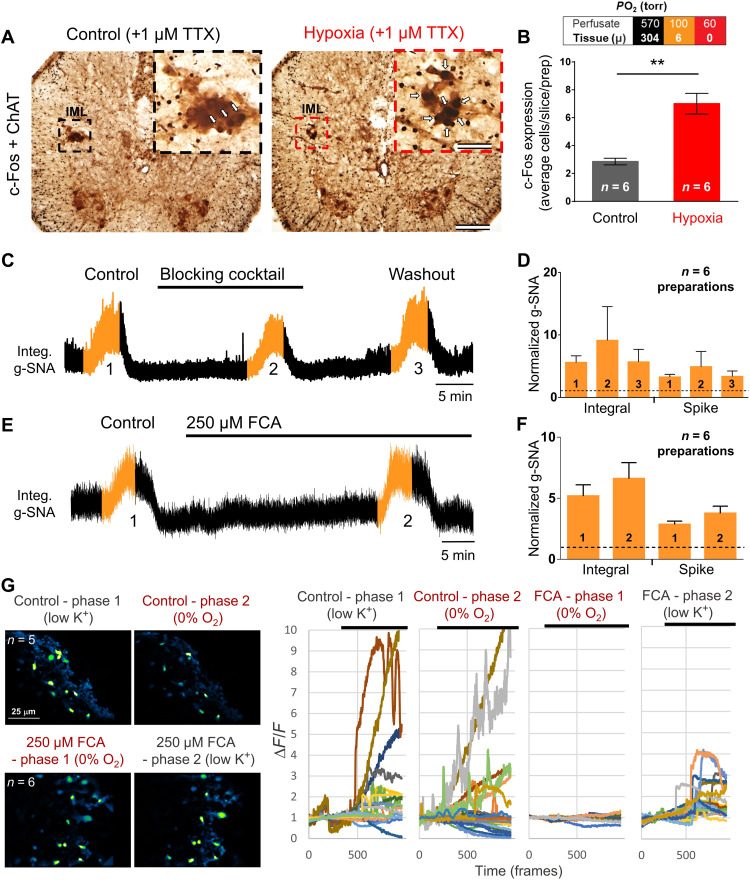
Endogenous oxygen sensitivity of SSPNs. (**A** and **B**) *c-fos* expression is induced in ChAT^+^ IML SSPNs by hypoxia (all experiments were performed in the presence of TTX to block network activity; unpaired *t* test with Welch’s correction, **P = 0.002; bars show means ± SEM). *c-fos* (nucleated; see arrows) and ChAT (diffuse, cytoplasmic) expressions were determined using immunohistochemistry. (**C** and **D**) Hypoxic responses of the in situ perfused spinal cord preparation persist under a blocking cocktail for common excitatory gliotransmitters and neurotransmitters [pyridoxalphosphate-6-azophenyl-2′,4′-disulfonic acid (PPADS) = 5 μM; 3,3′-diaminobenzidine (DAB) = 500 μM; oxamate = 2.5 mM; MRS2179 = 10 μM; suramin = 100 μM; trinitrophenol adenosine 5′-triphosphate (TNP-ATP) = 20 nM; d-lactate = 2 mM; DL-AP5 = 100 μM; 6-cyano-7-nitroquinoxaline-2,3-dione (CNQX) = 10 μM] (**E** and **F**) and under the astrocyte inhibitor fluorocitric acid (FCA; 250 μM). (**G**) Astrocyte Ca^2+^ responses to hypoxia in en bloc transverse thoracic spinal cord sections imaged using Oregon Green, demonstrating that responses to hypoxia are eliminated by FCA (low K^+^ excites astrocytes and inhibits neurons). As robust responses to hypoxia from the ventral root persisted when treated with FCA, these experiments demonstrate that the oxygen sensor likely resides in neurons but not in astrocytes. Note that a residual response to low K^+^ persists in FCA, suggesting that 250 μM FCA does not abolish all astrocyte function. Top and bottom: Two different preparations showing the *Z*-projection mean fluorescence with and without FCA, respectively. Amplitude and/or area under the curve indicates activity level.

To extend these findings to juvenile rats, we set about identifying oxygen-activated neurons within the IML using *c-fos* expression, indicative of recent neuronal activity. In these experiments, in situ thoracic spinal cords were used. Preparations were exposed to perfusate in which the *P*O_2_ was cycled between 570 and 60 torr, such that the tissue was exposed to three 5-min periods of anoxia (0.6 ± 2.0 torr) on a hyperoxic background (304.4 ± 58.0 torr). As with the en bloc preparation above, TTX was included in the perfusate to block network-dependent neuronal activity. Slides were stained using horseradish peroxidase with (*c-fos*) and without [choline acetyltransferase (ChAT)] nickel, such that the cytoplasm of ChAT-expressing cells appears brown, whereas the nucleus of *c-fos*-expressing cells appears black. Note that while spiking was blocked by TTX, TTX does not block increases in intracellular calcium, necessary for *c-fos* expression (*29*). *c-fos* expression in ChAT^+^ IML SSPNs was greater in hypoxia-perfused compared to time-control (hyperoxic) preparations (unpaired *t* test with Welch’s correction, *P* = 0.002; Fig. 6, A and B). These data again suggest that individual SSPNs are oxygen sensitive but do not fully exclude the possibility that their sensitivity originates from action potential–independent signaling from neighboring neurons or glia.

To begin to address the possibility that SOS originates from action potential–independent signaling from neurons or glia in the vicinity of the IML, we tested the hypoxic response of the splanchnic nerve with and without a “blocking cocktail.” This blocking cocktail was designed to target local fast excitatory synaptic transmission between neurons, as well as between neurons and astrocytes, and contained 100 μM suramin [adenosine 5′-triphosphate (ATP) receptor antagonist], 5 μM pyridoxalphosphate-6-azophenyl-2′,4′-disulfonic acid (PPADS)(P2 channel blocker), 10 μM MRS2179 (P2Y1 receptor antagonist), 20 nM trinitrophenol ATP (P2X2/3 blocker), 500 μM 1,4-dideoxy-1,4-imino-d-arabinitol (DAB) (blocker of astrocytic glycogen shunt activity, required for production of ATP), 2 mM d-lactate (l-lactate competitor), 2.5 mM oxamate (l-lactate dehydrogenase inhibitor), 100 μM 2-Amino-5-phosphonopentanoic acid (DL-AP5), and 10 μM 6-cyano-7-nitroquinoxaline-2,3-dione (glutamate receptor antagonists) (*30*, *31*). The prosympathetic effect of hypoxia was not blocked by this treatment (Friedman test, ANOVA multiple comparisons, *P* > 0.999; Fig. 6, C and D). Hence, intrinsic oxygen chemosensitivity—independent of primary synaptic inputs—likely resides, at least in part, within the IML.

### Role of astrocytes?

Recently, astrocytes were identified as putative brainstem oxygen sensors where they release ATP and stimulate P2Y receptors on respiratory neurons, reducing neuronal inhibition caused by central hypoxia (*21*). While the blocking cocktail is expected to block such signaling (*31*–*33*), the possibility remains that IML astrocytes detect *P*O_2_ changes and then signal to surrounding SSPNs via an exotic gliotransmitter. To further examine this, we incubated in situ thoracic spinal cord preparations with 250 μM gliotoxin fluorocitric acid (FCA), a concentration reported to be effective in similar preparations previously. Care was taken in preparing FCA to remove barium (*34*). A 40-min pretreatment with FCA had no effect on the SOS hypoxic response (paired *t* test, integral: *P* = 0.35 and spike: *P* = 0.15; Fig. 6, E and F). To ensure that IML astrocytes are inhibited by FCA at this concentration, we reverted to the neonatal transverse spinal cord preparation and used Oregon Green Ca^2+^ imaging in neonatal en bloc transverse thoracic spinal cord sections to visualize hypoxic responses of IML astrocytes. Hypoxia increased Ca^2+^ within IML astrocytes, but this increase was abolished by FCA (Fig. 6G). We conclude that while astrocytes may sense oxygen independently of neurons in the IML (or are recruited when neurons around them are activated by hypoxia), they do not appear necessary for IML hypoxic neuronal responses.

### Cellular mechanism of oxygen sensing

To get a line on the cellular oxygen sensing mechanism, we took a pharmacological and knockout (KO) approach, using the in situ thoracic spinal cord preparation to screen putative oxygen sensing mechanisms. We found that the SOS are critically dependent on neuronal nitric oxide synthase 1 (NOS1), reduced form of nicotinamide adenine dinucleotide (NADH) phosphate (NADP^+^) (NADPH) oxidase (NOX; predominantly NOX2), and reactive oxygen species (ROS)–dependent transient receptor potential (TRP) channels. Both NOS1 and NOX2 catabolize NADPH and are oxygen sensitive (*35*, *36*).

#### A role for NOS1

**Fig. 7.**
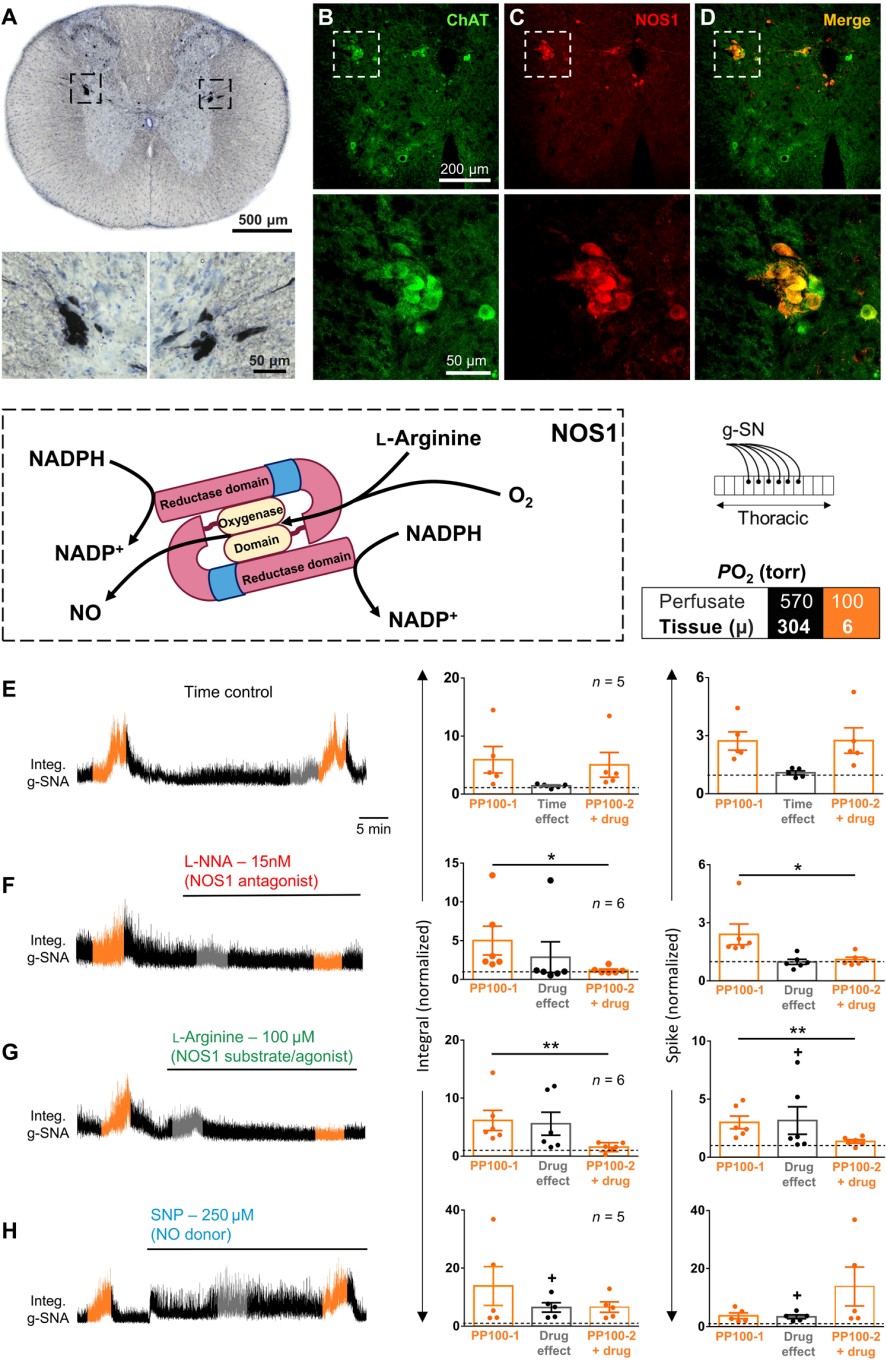
Role of NOS1 in SOSs. (**A**) Horseradish peroxidase immunohistochemistry for NOS1 in IML of rat thoracic spinal cord. (**B** and **C**) Double fluorescent immunohistochemistry for NOS1 (red) and ChAT (green) in mouse thoracic spinal cord. (**D**) Merged NOS1 and ChAT labeling. The IML (dashed squares) are magnified in the bottom panels. Note that NOS1 is expressed in all but most medial SSPNs as previously described, and the expression level may differ between SSPNs innervating different targets (*84*). (**E** to **H**) Box: Color-coding illustrates perfusate *P*O_2_ in torr. Left: g-SNA responses to hypoxia in rat in situ thoracic spinal cord preparations (see schematic). The hypoxic responses (perfusate and tissue *P*O_2_, 100 and ~6 torr, respectively) and drug effects were measured during the 1-min interval with the greatest response, illustrated in orange and gray, respectively. Right: Group data. (E) Time control; (F) L-NNA, NOS1 blocker; (G) l-arginine, NOS1 agonist; (H) SNP, NOS-independent NO donor. Dots show data from individual preparations; values exceeding dashed lines are greater than baseline. Bars show means ± SEM. Comparison between hypoxic responses before and with drug, *P < 0.05 and **P < 0.01; baseline effect of drug, +*P* < 0.05. Amplitude and/or area under the curve indicates activity level. Time scale in (E) applies to all traces. See table S2 for detailed statistics.

**Fig. 8.**
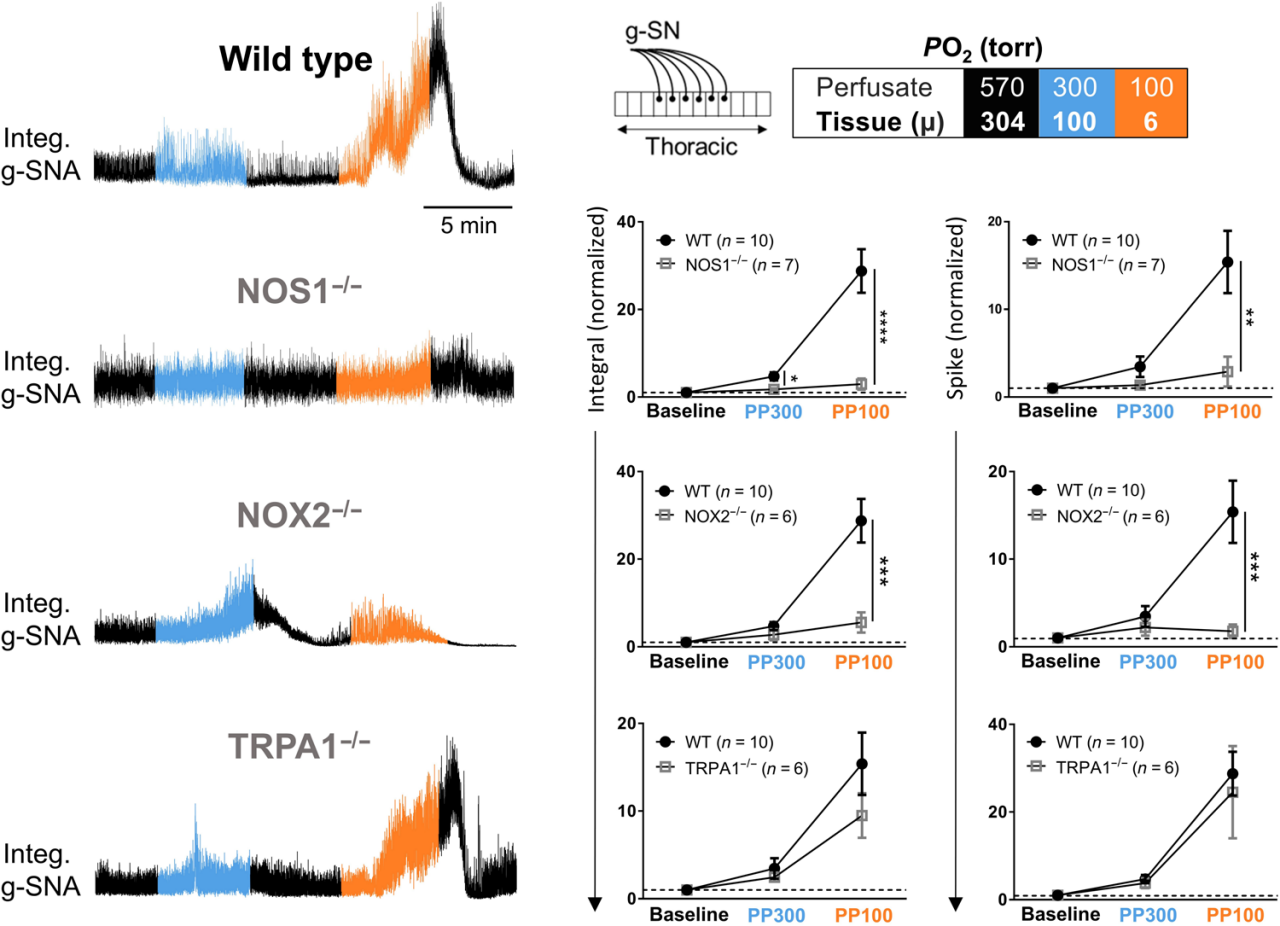
g-SNA responses to hypoxia in perfused in situ spinal cord preparation from wild-type and NOS1^−/−^, NOX2^−/−^, and TRPA1^−/−^ mice. Box: Color-coding illustrates perfusate *P*O_2_ in torr. NOS1^−/−^ and NOX2^−/−^, but not TRPA1^−/−^, mice have blunted responses to hypoxia compared to wild type (note that wild-type animals were pooled and replotted on each graph). Filled circles and open gray squares represent data (means ± SEM) from wild-type and KO mice, respectively. Amplitude and/or area under the curve indicates activity level. Statistical comparison performed with repeated-measures two-way ANOVA. See table S3 for detailed statistics.

NOS1 is highly expressed in ChAT^+^ IML neurons (Fig. 7, A to D) and oxygen sensitive across the entire physiological range (*K*_m_O_2_ of 350 μM; ~192-torr *P*O_2_), making it an attractive molecular candidate for the SOS. Others have reported that the IML NOS1 isoform critical for NADPH-diaphorase histochemistry is blocked by *N*^ω^-nitro-l-arginine (L-NNA) but not by the more conventional NOS1 inhibitor, 7-nitroindazole (*37*). Consistent with an essential role for NOS1 in spinal oxygen sensing, we found that hypoxic responses in the in situ preparation are abated by 15 nM L-NNA (Wilcoxon *t* test, integral: *P* = 0.031 and spike: *P* = 0.031; Fig. 7, E and F) and in NOS1^−/−^ (two-way ANOVA multiple comparisons, PP300 integral: *P* = 0.041, PP100 integral: *P* < 0.0001, PP300 spike: *P* = 0.78, and PP100 spike: *P* = 0.002; Fig. 8). l-Arginine (100 μM), which facilitates NOS production of NO, and the NO donor sodium nitroprusside (SNP; 250 μM) both increased baseline splanchnic nerve activity [l-arginine: one sample *t* test versus one, *P* = 0.07 (integral) and *P* = 0.031 (spike); SNP: one sample *t* test versus one, *P* = 0.03 (integral) and *P* = 0.021 (spike); Fig. 7, G and H], but, unexpectedly, only l-arginine blocked the hypoxic response [l-arginine: paired *t* test, *P* = 0.009 (integral) and *P* = 0.001 (spike); SNP: paired *t* test, *P* = 0.61 (integral) and *P* = 0.59 (spike)]. As NO production is proportional to *P*O_2_ (i.e., NO is the lowest when the SOSs are the most active) and NO stimulates splanchnic nerve activity, the above data are inconsistent with a direct role for NO in SSPN oxygen sensing. Instead, we propose a novel mechanism in which NOS1 acts as an oxygen-dependent sink for NADPH, serving to determine the availability of NADPH for other NADPH-dependent molecules. That is, the lower the *P*O_2_, the greater the availability of NADPH for another molecule.

#### A role for NOX2

**Fig. 9.**
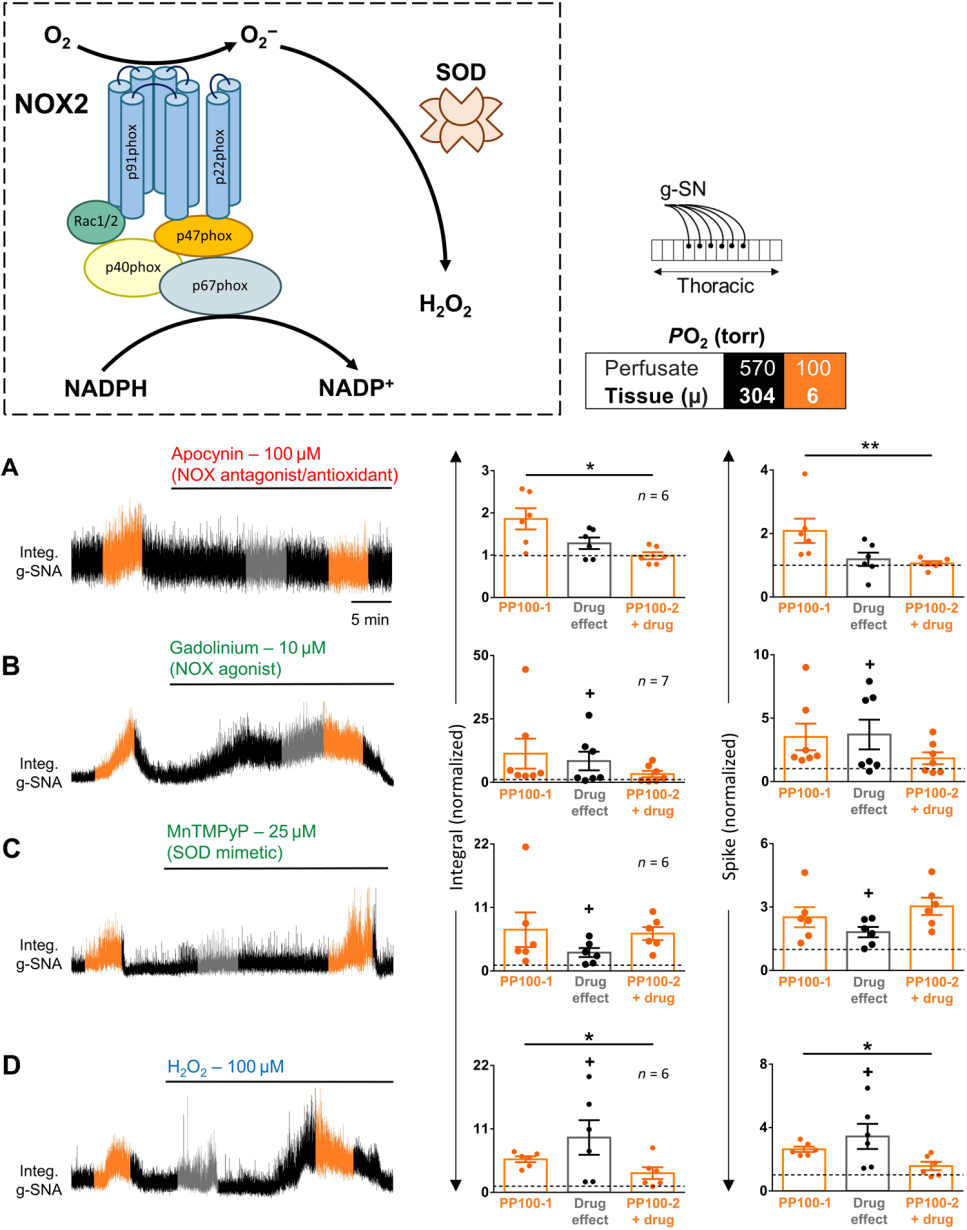
Role of NOX and ROS in SOSs. Box: Color-coding illustrates perfusate *P*O_2_ in torr. Left: g-SNA responses to hypoxia in rat in situ thoracic spinal cord preparations (see schematic). The hypoxic responses (perfusate and tissue *P*O_2_, 100 and ~6 torr, respectively) and drug effects were measured during the 1-min interval with the greatest response, illustrated in orange and gray, respectively. Right: Group data. (**A**) Apocynin, NOX inhibitor; (**B**) gadolinium, NOX agonist; (**C**) MnTMPyP, SOD mimetic; (**D**) H_2_O_2_, ROS. Dots show data from individual preparations; values exceeding dashed lines are greater than baseline. Bars show means ± SEM. Amplitude and/or area under the curve indicates activity level. Comparison between hypoxic responses before and with drug, *P < 0.05 and **P < 0.01; baseline effect of drug, +*P* < 0.05. Time scale in (A) applies to all traces. See table S2 for detailed statistics.

On the basis of the above model, we investigated a role for NOX. NADPH and O_2_ are catabolized by NOX into NADP^+^ and ROS such as superoxide ^·^O_2_^−^ and [via superoxide dismutase (SOD)] H_2_O_2_ (Fig. 9). ^·^O_2_^−^ and H_2_O_2_ are highly potent signaling molecules providing a mechanism to activate cells. Consistent with a role for NOX and ROS, we used the in situ spinal cord preparation to demonstrate the complete inhibition of SOS by a broadband NOX inhibitor/antioxidant (100 μM apocynin; paired *t* test, integral: *P* = 0.014 and spike: *P* = 0.007), and their masking by NOX agonist (10 μM Gd^3+^; Wilcoxon matched-pairs signed-rank test, integral: *P* = 0.58 and spike: *P* = 0.47) (Fig. 9, A and B) (*38*). Using this preparation, we also found that the mimicking (i.e., excitation of SSPNs; one sample *t* test versus one, integral: *P* = 0.038 and spike: *P* = 0.021) and amplification of the hypoxic response by SOD mimetic 25 μM MnTMPyP (Mn(III)tetrakis(1-methyl-4-pyridyl)porphyrin) (paired *t* test, integral: *P* = 0.66 and spike: *P* = 0.49) are in contrast to the mimicking and subsequently masking of the hypoxic response by exogenous 100 μM H_2_O_2_ (paired *t* test, integral: *P* = 0.037 and spike: *P* = 0.038; Fig. 9, C and D). Involvement of NOX2 is supported by the partial abrogation of the hypoxic response in NOX2^−/−^ (two-way ANOVA multiple comparisons, PP300 integral: *P* = 0.16, PP100 integral: *P* = 0.0002, PP300 spike: *P* = 0.48, and PP100 spike: *P* = 0.0004; Fig. 8). We specifically focused on NOX2 because of its low *K*_m_O_2_ compared to most other oxygen-sensitive molecules, indicating its ability to function in the presence of residual levels of O_2_. Nonetheless, the contrast between the complete inhibition of the SSPNs’ hypoxic response by a broadband NOX inhibitor/antioxidant and the partial abrogation in NOX2^−/−^ may suggest the involvement of multiple NOX species and/or other NADPH-dependent molecules.

#### Effectors

**Fig. 10.**
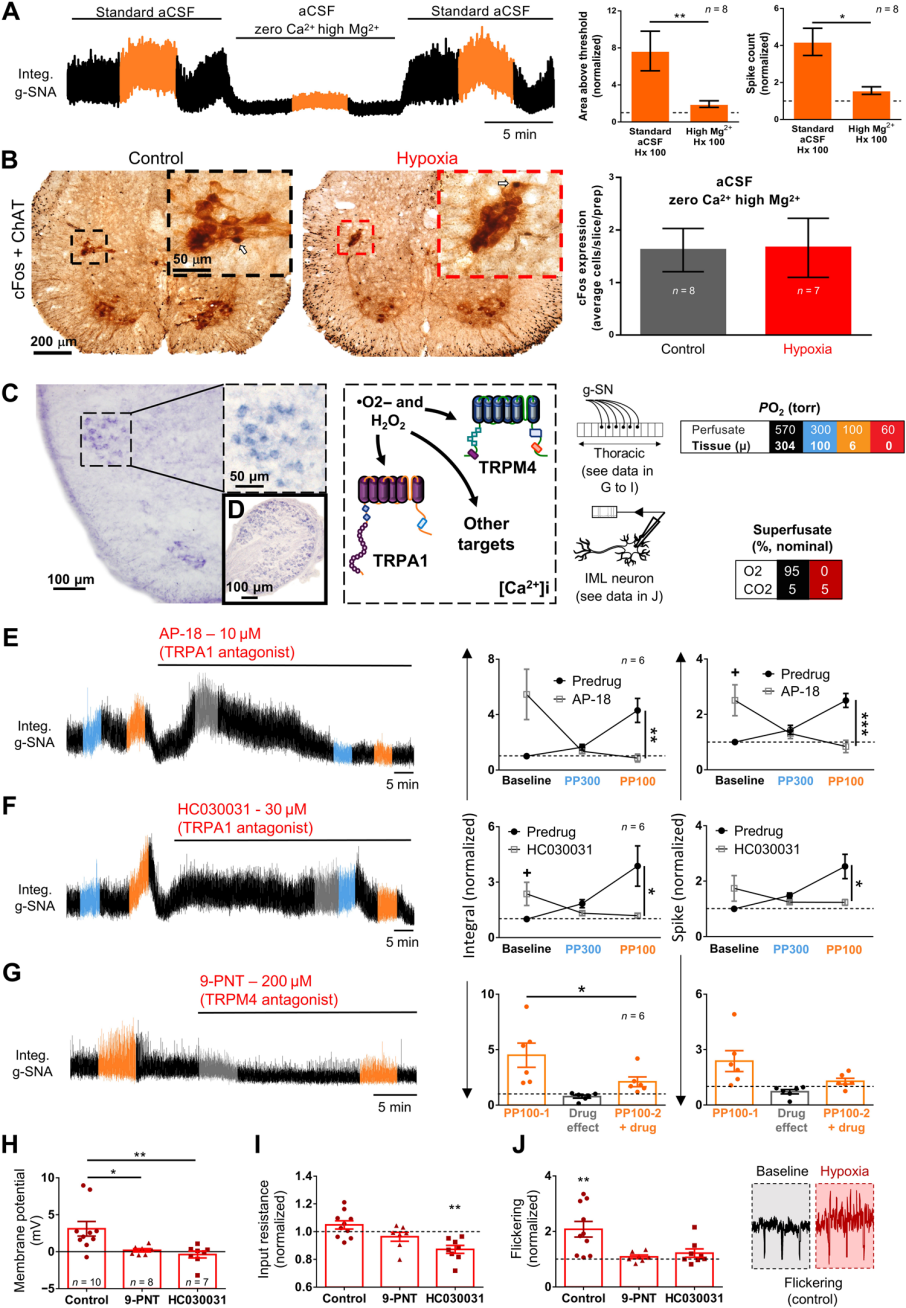
Role of Ca^2+^ and TRP channels in SOSs. (**A** and **B**) Abatement of hypoxia-induced sympathetic nerve activity (g-SNA; Wilcoxon signed-rank test, integral: *P* = 0.008 and spike: *P* = 0.015) and *c-fos* production (unpaired *t* test, *P* = 0.95) in rat in situ preparation when Ca^2+^-dependent synaptic transmission is suppressed. (**C**) In situ hybridization (ISH) of TRPA1 expression in a thoracic segment of P2 rat spinal cord and (**D**) dorsal root ganglion (positive control). (**E** to **G**) Effect of TRP channel blockers on SOSs in rat in situ preparation. g-SN activity with perfusate *P*O_2_ of 300 torr (tissue, ~100 torr; blue), 100 torr (tissue, ~6 torr; orange), and during drug (gray). Note that two levels of *P*O_2_ were used to probe TRPA1 involvement because others report differential effects at different *P*O_2_ (*50*, *85*). (**H** to **J**) SSPN membrane potential (whole-cell recording) increases during hypoxia (dark red) in control group but not after treatment with HC030031 (*P* = 0.003) and 9-phenanthrol (9-PNT) (*P* = 0.03) in en bloc preparation. (I) Input resistance decreased by HC030031 (*P* = 0.003), indicating involvement of calcium-dependent outward currents. (J) Membrane potential flickering increased under control conditions under hypoxia (see inlay) but not in presence of HC030031 or 9-PNT, indicating hypoxic recruitment of ion channels (*P* = 0.004). Bars show means ± SEM. Comparison between hypoxic responses before and with drug, *P < 0.05, **P < 0.01, and ***P ≤ 0.001; baseline effect of drug, +*P* < 0.05. See tables S2 and 4 for detailed statistics.

Recall that in TTX, whole-cell recordings revealed a small but significant change in membrane potential without a change in input resistance during hypoxic challenge (Figs. 5C and 10J). This finding suggests that changes in intracellular calcium may play an important role in oxygen-dependent regulation of cell firing. To further explore this idea, we perfused the in situ preparation with high Mg^2+^/zero Ca^2+^ to prevent Ca^2+^ entry and deplete intracellular Ca^2+^ stores. Under these conditions, hypoxia-induced g-SN responses and *c-fos* staining were all but abolished [comparison of hypoxic response with and without high Mg^2+^/zero Ca^2+^; integral: Wilcoxon signed-rank tests on g-SN, *P* = 0.008 (Fig. 10A, left graph); spike: Wilcoxon signed-rank test on g-SN, *P* = 0.015 (Fig. 10A, right graph); *c-fos*–labeled cells: unpaired *t* test with Welch’s correction, *P* = 0.95 (Fig. 10B)].

In addition, consistent with an important role for Ca^2+^ signaling, we found that two broad spectrum and nonspecific drugs that are reported to block TRP channels and disrupt intracellular Ca^2+^ regulation, 100 μM 2-aminoethoxydiphenyl borate (2-APB) (integral: paired *t* test, *P* = 0.0006; spike: Wilcoxon test, *P* = 0.3), and 100 μM flufenamic acid (Wilcoxon test, integral: *P* = 0.031 and spike: *P* = 0.031) also perturbed oxygen sensing in the thoracic in situ spinal cord preparation (fig. S5). Therefore, we tested whether ROS and calcium-sensitive TRP channels, TRPA1 and TRPM4, are important downstream components of the SOS mechanism. Using in situ hybridization (ISH), we found that TRPA1 expression is concentrated in the IML (Fig. 10C). Moreover, two TRPA1 antagonists (10 μM AP-18 and 30 μM HC030031) provoke g-SN activity and abolish the hypoxic response (e.g., on spike, repeated-measures two-way ANOVAs, *P* < 0.05; see table S2) in the thoracic in situ spinal cord preparation (Fig. 10, E and F). Bath-applied HC030031 (10 μM) also suppressed hypoxia-induced membrane depolarization (Kruskal-Wallis test, *P* = 0.003) and flickering (one sample *t* test versus one, *P* = 0.19) of IML neurons recorded with whole-cell patch in TTX-treated en bloc transverse thoracic spinal cord sections (Fig. 10, H and J). Notwithstanding the complete abolition of oxygen sensing with TRPA1 antagonists, we found that the SOS was preserved in TRPA1^−/−^ mice (Fig. 8). These apparently conflicting results suggest a role for compensatory or redundant mechanisms in transducing ROS to g-SN excitability. H_2_O_2_ is reported to stimulate TRPM4 preventing channel desensitization (*39*). In this light, we also investigated a role for TRPM4 using the antagonist 9-phenanthrol (9-PNT; 200 μM). 9-PNT abolished hypoxic g-SN responses in situ (paired *t* test, integral: *P* = 0.047 and spike: *P* = 0.12). 9-PNT also abolished SSPN single-cell responses in en bloc preparations, abolishing hypoxia-induced single-cell membrane depolarization (Kruskal-Wallis test, *P* = 0.032) and flickering (one sample *t* test versus one, *P* = 0.25) (Fig. 10, H and J). Given the broad effects of ROS in cell signaling and the likelihood that the antagonists used above have additional off target effects, we consider it likely that one or more other effector molecules are at play in transducing elevated ROS to SSPN excitability.

## DISCUSSION

Our data provide evidence that SSPNs in the IML constitute high-fidelity SOSs and suggest that the SOSs have life-saving capability by modulating phrenic activity, including the initiation of gasps, in the absence of brainstem asphyxia. Moreover, on the basis of pharmacology and KO mice data, we propose a novel oxygen sensing mechanism, assigning a new functional role for NOS1 in which NO production is secondary to NADPH substrate catabolism.

### Importance of NOS1 and NOX2

**Fig. 11.**
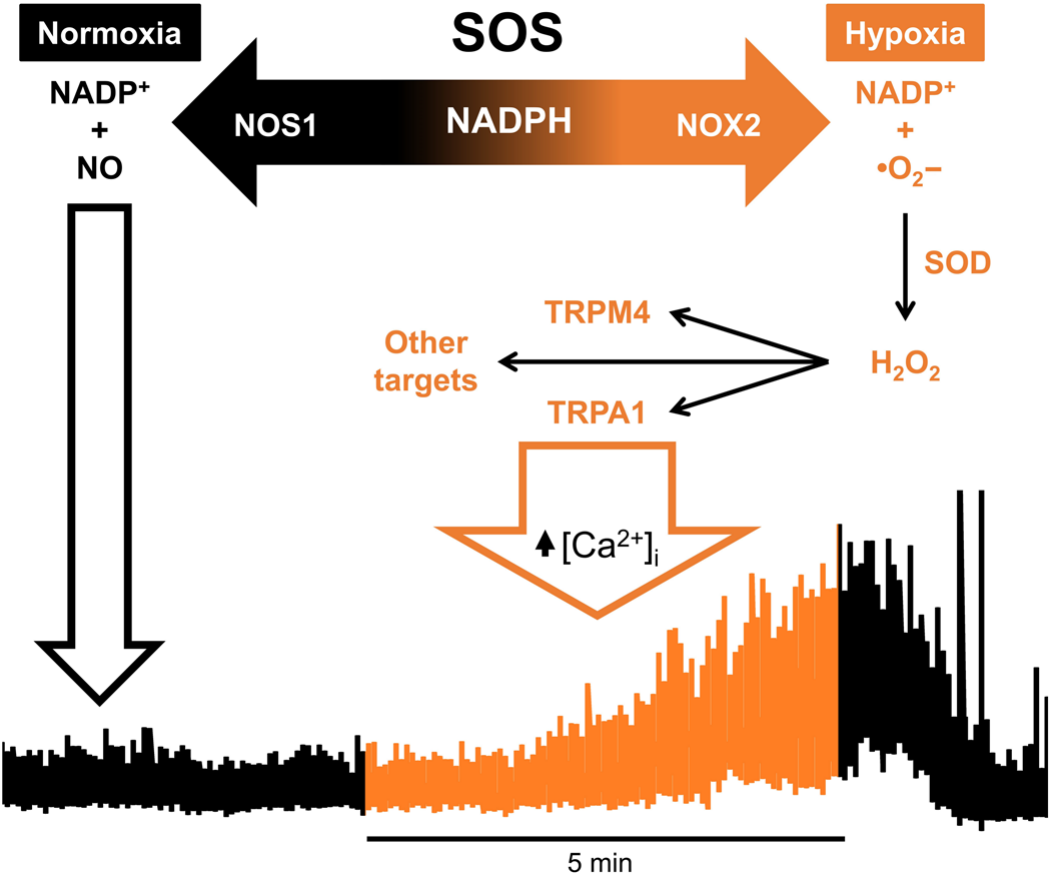
Model of SOS oxygen sensing mechanism. Two enzymes NOS1 (neuronal NOS) and NOX2 compete for a common substrate NADPH. NOS1 is highly abundant in the IML (Fig. 7A) and serves as a *P*O_2_-dependent sink for NADPH. When *P*O_2_ falls, the NADPH availability increases, increasing NOX2 activity and generating superoxide (^·^O_2_) and H_2_O_2_. ROS activate multiple receptors including TRPA1 and TRPM4, increasing intracellular calcium and SSPN activity (illustrated by trace). This model implies that a residual amount of oxygen is required to sustain NOX production of ROS and SOS responses; even when zero tissue *P*O_2_ is maintained, the SOSs fail.

Our data demonstrate that l-arginine (which increases NOS1 activity and NO production) and NO donor SNP have qualitatively different effects on the hypoxic response. As NOS1 abrogation (with L-NNA or genetic deletion) and acceleration (with l-arginine) both compromise oxygen sensitivity, these data suggest that launching sympathetic responses to hypoxia involves the increased availability of NADPH. Thus, we propose that the lower the *P*O_2_, the greater the availability of NADPH for NOX2, the faster the production of ROS, and the greater the activity of ROS-excited SSPNs (Fig. 11). The high concentration of NOS1 in the SSPNs plays an important role in this model, ensuring that NADPH only accumulates when activity of NOS1 is limited by low O_2_. This oxygen sensing model is also consistent with the relative *K*_d_ values of NOS1 and NOX2 for NADPH [2.5 and 50 μM, respectively (*40*, *41*)] and the relative *K*_m_O_2_ of NOS1 and NOX2 (192- and 5- to 22-torr *P*O_2_, respectively) as further detailed below.

NADPH serves as substrate for HO and NOX, both proposed as candidate oxygen sensing molecules in the carotid body (*1*, *2*, *42*–*44*) and RVLM (*10*). HO may provide an additional oxygen-sensitive mechanism for catabolizing NADPH in the spinal cord, but unlike NOS1, HO is widely distributed in the spinal cord and not concentrated in the IML. Further, HO does not appear to be critical for SOS function because the suppression of NOS1 or NOX2 activity is pharmacologically or genetically sufficient to abrogate oxygen sensing. The high expression levels of NOS1 in SSPNs and its relatively high *K*_m_O_2_ (350 μM; O_2_, ~192 torr) and low *K*_d_NADPH (2.5 μM) compared to HO [*K*_m_O_2_, 28 to 77 μM; O_2_, ~42 torr (*45*); *K*_d_NADPH, 6.1 ± 0.4 μM (*46*)] suggest that NOS1 is likely more effective at catabolizing NADPH in the SSPNs when tissue oxygenation is normal [*P*O_2_, ~30 to 48 torr (*25*)].

According to the above model of oxygen sensing, only when oxygen levels fall does NADPH become available for NOX2, increasing ^·^O_2_^−^ and/or H_2_O_2_. A previous report by Su *et al*. (*47*) suggests that NO is obligatory for SSPN activity, raising the possibility that the excitability of SSPNs is proportional to NO production. Although this may explain some of our observations, our data are not entirely consistent with an obligatory and/or excitatory role of NO for SSPN activity. For example, despite increasing NO production assumed to occur with l-arginine, l-arginine suppresses the hypoxic response. In addition, while the hypoxic response is significantly abated in L-NNA–treated and NOS1^−/−^ spinal cords, much of the transient response to ischemia remains (fig. S6). Nonetheless, Su *et al*. (*47*) observations can be easily accommodated in our model by assuming that during SSPN responses to hypoxia, sufficient oxygen is available to support residual NOS1 activity, just not at the level of activity required to deplete available NADPH. Similarly, the low *K*_m_O_2_ of NOX2 (5- to 22-torr *P*O_2_) indicates that NOX2 will continue to produce ROS at oxygen levels well below that required to sustain activity of other oxygen-dependent enzymes, including NOS1. Ultimately, however, as tissue *P*O_2_ approaches zero, even NOS1 and NOX2 will cease to function, and ROS production will terminate. Thus, according to our model (and entirely consistent with our experimental observations), SSPN activity induced by hypoxia is not sustained once tissue anoxia is reached.

### Model limitations

On the basis of the long history of research into oxygen sensing in the carotid body, we might expect that the above model is only a starting point in understanding the mechanism used by the SOS. For example, only recently have high levels of gene expression for protein isoforms in the carotid body been identified that appear to explain its exquisite sensitivity to oxygen (*2*). In contrast, our model already draws on the high expression levels of NOS1 and TRPA1 in SSPNs, but we have not examined whether the SSPN isoform for NOS1 is specific to the spinal cord. For example, we note that NOS1 is also present in the phrenic motor nucleus (cervical ventral horn) and regulates phrenic motor facilitation by a 5-Hydroxytryptamine 2B receptor– and NOX-dependent mechanism (*48*). Therefore, a parallel mechanism may exist within the phrenic motor nucleus.

Similar to NOS1, we show that TRPA1 is also strongly expressed in SSPNs. Our model assigns TRPA1 an important role in sensing ^·^O_2_^−^/H_2_O_2_ with high expression levels likely helping to enhance oxygen sensitivity. An important role for TRPA1 is supported by the loss of oxygen sensing in the presence of widely used and structurally distinct antagonists AP-18 and HC030031. However, oxygen sensing persisted in TRPA1^−/−^ mice. While HC030031 may have some off-target effects on sodium channels, we have not found any reports of offsite drug effects of AP-18 that would compromise our findings. Therefore, compensation and redundancy between effector mechanisms are likely. In search of other effector mechanisms, we also identified an important role for another ^·^O_2_^−^/H_2_O_2_-sensitive molecule in the SOS, namely, TRPM4, and do not exclude the possible involvement of other ^·^O_2_^−^/H_2_O_2_-sensitive molecules. Additional ^·^O_2_^−^/H_2_O_2_-sensitive candidates may include those involved in regulating intracellular calcium stores, such as inositol 1,4,5-trisphosphate receptor (IP3R) (*49*).

TRPA1 has been implicated in oxygen sensing in astrocytes, vagal ganglia, and other systems through direct oxygen-dependent mechanisms involving prolyl hydroxylases and cysteine-mediated oxidation (*50*). These mechanisms are thought to function at distinct and nonoverlapping levels of *P*O_2_. An essential nonredundant role for TRPA1 as the oxygen sensor in SSPNs is not supported by the persistency of a robust SSPN hypoxic response in TRPA1^−/−^ as discussed above. Nonetheless, our current data lack the resolution to exclude TRPA1 (and/or hitherto unfound additional molecules and mechanisms) having a direct role in oxygen sensing over narrow ranges of *P*O_2_ (*50*). Given that some residual sensitivity of SSPNs to hypoxia persists in NOS1^−/−^ and NOX2^−/−^, the NOS1-NOX2 model we propose is likely one of several ways in which the SSPNs sense and respond to hypoxia.

### Experimental limitations in determining SOS sensitivity

Our work has demonstrated spinal oxygen sensitivity in adult in vivo, juvenile in situ, and neonatal rostral and caudal thoracic en bloc preparations, from rats and mice. In our analysis of mechanism, we assume that the same oxygen sensing mechanisms is at play across these preparations, although the level, gradient, and dynamics of tissue *P*O_2_ are likely to be quite different. For example, in the spinalized in vivo preparation (the only preparation that uses hemoglobin as an oxygen carrier), we compared g-SN responses to SaO_2_ changes induced by changing inspired O_2_, whereas in the other preparations, we changed the *P*O_2_ used to equilibrate perfusate. In addition, in each preparation, the normal supply of oxygen to the spinal cord was disrupted/replaced in different ways. In the in vivo preparation, local spinal vasculature at the site of transection (T2 and T3) was disrupted catastrophically. This local disruption may have altered tissue *P*O_2_ throughout the remaining spinal cord. At the other extreme, we measured the *P*O_2_ threshold of ventral root activity in en bloc transverse thoracic spinal cord section preparations. However, the oxygen sensitivity of SSPNs in these preparations is likely to be less than in a more intact network owing to loss of intersegmental ramifications and SSPN interconnectivity. In addition, tissue *P*O_2_ decreases rapidly with depth in en bloc preparations such that different SSPNs within spinal segment will experience different *P*O_2_. In comparison, in the in situ preparation, much of the IML network is intact, and the tissue is likely to be oxygenated more uniformly. Conversely, in the in situ preparation, our attempts to directly measure spinal cord tissue *P*O_2_ failed because of the disruption of the local vasculature caused by the dorsal laminectomy required to place the oxygen electrode. Ultimately, we overcame this issue by measuring tissue *P*O_2_ in the brainstem as a surrogate for spinal cord *P*O_2_ because, unlike the spinal cord, which is perfused via penetrating vessels from both ventral and dorsal surfaces, the majority of brainstem circulation originates ventrally. In doing so, we introduce a new caveat, the assumption that the packing density of neurons, degree of vascularization, and autoregulatory properties of the brainstem and spinal cord are the same (*51*, *52*). In summary, in our study of the SOS, we have used multiple types of preparations in mice and rats each having their own caveats. Nonetheless, that SOS are present across these preparations speaks to their resilience and physiological importance.

## IMPLICATIONS

In identifying an important role for SSPNs and a novel NOS1-NOX-ROS mechanism in CNS oxygen sensing, our data raise new questions about the relative efficacy, sensitivity, and interactions among oxygen chemoreceptors (*53*). In the intact in vivo system, other potential oxygen sensors include carotid body glomus cells and astrocytes and/or neurons of the hypothalamus, NTS, pre-Bötzinger complex, retrotrapezoid nucleus (RTN), and C1 (*1*, *2*, *7*–*10*, *23*). Of the central sites, oxygen sensing by RTN astrocytes requires TRPA1, but only the SSPNs appear to involve NOS1 (*23*, *54*). We also note that microglia may be candidate oxygen sensors, although we did not explicitly test this here.

Among the above sites, the carotid bodies have the greatest oxygen sensitivity and efficacy in driving cardiorespiratory activity and thus require special consideration. Acute oxygen sensing by the carotid bodies likely involves glomus cell mitochondria that use a specialized electron transport chain (ETC). Accordingly, glomus cell mitochondria likely require a higher *P*O_2_ to maintain an efficient ETC than do mitochondria in other cell types. The molecular cause of this differential sensitivity to *P*O_2_ is under active investigation but may involve modulation by gaseous signaling molecules (including NO) and/or a unique combination of mitochondrial subunits (*55*). Regardless, slight falls in oxygen are believed to cause a backup of electrons, resulting in production of ROS/NADH, that then signal to the rest of the glomus cell to trigger neurotransmitter release. How ROS/NADH activates glomus cells is unknown but likely also involves TRP channels to some degree (*2*, *56*). Despite involving very distinct mechanisms of ROS production between the carotid bodies and SOS (only oxygen sensing by the SOS requires NOX2), their sensitivity between 60- and 300-torr *P*O_2_ in arterially perfused preparations appears to be somewhat similar. Consequently, the SOS and the carotid bodies may jointly contribute to cardiorespiratory control in the face of hypoxic stress.

More work is required to determine the clinical implications of the SOS, but the SOS and carotid bodies may jointly contribute to hypoxic responses encountered during mild cerebral vascular impairment, obesity, atherosclerosis, diabetes, asthma, chronic obstructive pulmonary disease, sleep apnea, and acute mountain sickness, all of which are associated with increased sympathetic activity. While not the focus of this study, we also note that severe intermittent hypoxia–induced sympathetic hyperactivity and hypertension can occur with subdued carotid body activity, suggesting a central site of plasticity that may involve increased SSPN tone (*57*). Increased SSPN tone is associated with ^·^O_2_^−^/H_2_O_2_ production (*58*), and ROS and NADPH are implicated in intermittent hypoxia–induced cardiorespiratory plasticity of sensory and motor activity (*48*, *57*). Thus, the effects of intermittent hypoxia on SSPN oxygen sensing also warrant study.

As respiratory and sympathetic responses resulting from asphyxia-like conditions, including gasps, were enhanced by the SOS, the SOS may also serve to prolong survival mechanisms triggered in response to severe CNS hypoxia caused by acute ischemia, hemorrhage, and/or sudden cardiorespiratory arrest. As severe hypotension is expected to reduce flow to (and therefore oxygenation of) the CNS, sympathoexcitation following acute spinal cord injury (SCI) and stroke may also be explained by the direct impact of acute injury and stroke on spinal oxygen supply (*59*). Similarly, the SOS are likely important after high SCI when the classic arterial baroreflex is incapacitated; the profound hypotension experienced by many patients with SCI may trigger SOS-mediated pressor activity preventing further reduction in blood pressure.

In summary, we demonstrate novel molecular and cellular mechanisms that are of sufficient sensitivity and efficacy to contribute to the brain’s primary oxygen defensive mechanisms. Thus, the SOS may help underpin the selfish brain hypothesis, which posits that a primary role of increased sympathetic nerve activity and resulting hypertension is to maintain adequate cerebral vascular perfusion (*60*). Our data strongly indicate SSPNs as the oxygen sensors. SSPNs are well suited to this task: They are highly oxygen sensitive, the most direct and least synaptic dependent of any central sympathetic relay and innervate practically every end organ and blood vessel in the body.

## MATERIALS AND METHODS

All procedures were approved by University of Calgary, Showa University, and The Florey Animal Care Committees and were in accordance with national and international guidelines. Some data were excluded from this report. Only the dataset from the most comprehensive blocking cocktail is included; several earlier datasets using subsets of the blocking cocktail are not only included but were also without effect. One dataset using *c-fos* was excluded because multiple attempts at independently verification failed. Preparations were also excluded on occasion for technical reasons. For example, preparations were excluded if nerve and/or cell recordings were lost before protocol completion or in protocols with an initial hypoxic challenge that failed to yield a robust hypoxia response. In total, we report data from 248 rats and 30 mice. All experiments were performed in Calgary (altitude of 1045 m) with gases reported in partial pressured and adjusted for daily changes in atmospheric pressure, unless otherwise stated.

### In vivo anesthetized preparation

The effects of hypoxia on sympathetic activity in spinalized, vagotomized, carotid body, and aortic arch–denervated animals were assessed in urethane-anesthetized (10% urethane, 1.3 g/kg, intraperitoneal) Sprague-Dawley male rats (means ± SEM, 280.2 ± 10.5 g; Charles River, Canada) (Fig. 1, A to D). Surgery commenced once animals were unresponsive to paw pinch. Blood oxygen saturation (SO_2_) was monitored throughout the experiment using a hindlimb pulse oxymeter (Model 8500, Nonin Medical, USA). The external jugular vein (for the administration of fluids) and the right femoral artery (for recording blood pressure) were cannulated. The trachea was cannulated, and the preparation was artificially ventilated with a gas having a *P*O_2_ of 200 torr (balanced N_2_), paralyzed with rocuronium bromide [Zemuron; initial dose: 2mg/kg, intravenously (i.v.) and then 1 mg/kg per hour, i.v.) and vagotomized. The carotid sinus nerve and cardiac depressor nerves were also cut bilaterally. Denervation was performed by A.R. who has 20 years of experience in recording from the carotid sinus nerve; we did not do NaCN bolus injections due to reports that responses persist despite carotid sinus nerve denervation (*61*). A small laminectomy was performed to expose the spinal cord at the level of T2 and T3. Sympathetic nerve activity was recorded from the left splanchnic nerve, which was exposed and placed on hook electrodes within an oil bath constructed in the retroperitoneal cavity. Recordings were made before and after two segments of the spinal cord (T2 and T3) were removed.

Neural activity was amplified, filtered (300-Hz low cutoff and 5-kHz high cut-off), rectified, and integrated (200-ms time constant). Raw and integrated recordings were stored on a computer using an analog to digital board and data acquisition software (CED Ltd., UK) at a sampling rate of 5 kHz. Analysis was performed offline with custom software. To illustrate change in g-SN burst activity with inspired *P*O_2_, we averaged 10 rectified and integrated burst pairs recorded when inspired *P*O_2_ was either 200 or 600 torr. Figure 1B (inlay) shows an overlay of the two averaged traces.

### In situ preparations

Three in situ preparations based on the working heart-brainstem preparation were used in this study (*62*). These preparations lack haemoglobin to carry oxygen; the only oxygen in the aCSF is that, which is dissolved. Consequently, even when the *P*O_2_ of the aCSF is very high, the actual arterial oxygen content entering the preparation is extremely low compared to an artery in an intact animal containing oxygenated blood. As neural tissue has a high metabolic rate and consumes oxygen rapidly, *P*O_2_ falls precipitously from vessels across the tissue. Thus, in the in situ preparation, an arterial *P*O_2_ of 200 torr, for example, results in a mean tissue *P*O_2_ of ~48 torr. The following procedures are common to all three in situ preparations. Before surgery, animals were anesthetized deeply in an atmosphere saturated with isoflurane (1-chloro-2,2,2-trifluoroethyl-difluoromethylether). Once an animal failed to respond to noxious pinch to the tail or hind paw, it was transected below the diaphragm. The rostral half, including the thoracic and first lumbar segments of the spinal cord, was transferred to ice-cold (~4°C) perfusate and immediately decerebrated. The perfusate contained 115 mM NaCl, 4 mM KCl, 1 mM MgSO_4_, 24 mM NaHCO_3_, 1.25 mM NaH_2_PO_4_, 2 mM CaCl_2_, 10 mM d-glucose, and 12 mM sucrose. The subsequent dissection was performed in ice-cold perfusate equilibrated with 95% O_2_/5% CO_2_. Routinely, neural activity was amplified, filtered (300-Hz low cutoff and 5-kHz high cutoff), displayed on an oscilloscope, rectified, and integrated (200-ms time constant; MA-821/RSP Moving Averager, CWE Inc.). Raw and integrated recordings along with pump speed were stored on a computer using an analog to digital board (Digidata 1322A, Axon Instruments) and data acquisition software (Axoscope 9.0) at a sampling rate of 5 kHz. Most figures illustrating responses of individual animals show rectified and integrated data; increased activity occurs when peak amplitude or area under the curve increases, depending on the baseline level of activity and rate of response. Analysis was performed offline with custom software (see below).

#### Perfused in situ brainstem–spinal cord preparation

To determine CNS tissue *P*O_2_ in perfused preparations, we used the in situ dual perfused preparation (Fig. 2, A to C) (*63*). In this decerebrated, vagotomized, and eviscerated preparation, the CNS including brainstem, cervical, and thoracic spinal cord is perfused retrogradely via the descending aorta (central perfusate equilibrated with *P*O_2_ of 570 torr and *P*CO_2_ of 40 torr); the carotid bodies are perfused with a separate line via the common carotid arteries (carotid body perfusate equilibrated with *P*O_2_ of 100 torr and *P*CO_2_ of 35 torr). Following surgery, this in situ brainstem–spinal cord preparation was pinned dorsal up with the cranium and cerebellum removed to expose the fourth ventricle. CNS perfusion pressure was gradually increased to 90 mmHg, as the preparation was warmed to 33° to 34°C with pressure maintained at this level using a computer-controlled feedback system. Hook electrodes were used to record activity in both the phrenic nerve and g-SN to ensure preparation viability.

##### In situ tissue PO_2_ measurements

We used the accessibility of the brainstem in the dual perfused preparation to measure CNS tissue *P*O_2_ in situ (Fig. 2, A to C). A Clark-style *P*O_2_ microelectrode (tip diameter of 25 μm to reduce tissue damage; time constant is less than 1 s) and a polarographic amplifier (Unisense A/S, Denmark) were used for this task. The output of the polarographic amplifier was digitized at 5 kHz using an analog to digital converter (Digidata 1322A, Axon Instruments) and recorded (Axoscope 9.0). The electrode was calibrated (i) once at 33°C in physiological saline and found to have linear responses between 0- and 600-torr *P*O_2_ and (ii) throughout experiments in tonometers bubbled with 100% O_2_ and 100% N_2_. After calibration, the electrode was attached to a stereotaxic apparatus that included a micromanipulator and the tip positioned directly above the ventrolateral medulla. The electrode was lowered until the tip touched the surface of the fourth ventricle; this was confirmed by sudden change in the signal from the electrode, which included loss of pump-associated fluctuations in *P*O_2_. One hundred–micrometer steps were used to penetrate the tissue and carry the tip of the electrode to a depth of 400 to 800 μm. In some locations, readings dropped progressively to zero over 10 min without the preparation demonstrating gasping or failed to stabilize, likely reflecting a blocked electrode tip. In these cases, the electrode was withdrawn and repositioned. Once a stable tissue *P*O_2_ was obtained, the *P*O_2_ of the gas used to equilibrate the perfusate was decreased (from 570 to 400, 300, 200, 100, 60, and 0 torr) in 5-min steps. Each step caused tissue *P*O_2_ to decay exponentially, either resulting in a tissue *P*O_2_ of 0 or approaching a nadir after 5 min. In the latter case, the tissue *P*O_2_ at the 5-min mark was determined by curve-fitting an exponential to eliminate the effects of sudden transients.

#### Novel perfused in situ thoracic spinal cord preparation

For these preparations, we used Sprague-Dawley male juvenile rats (P2 and 4 to 6 weeks old, 80 to 120 g; Charles River, Canada) or mice (6 to 12 weeks old, 20 to 30 g; wild-type C57Bl/6 supplied by P. Whelan, University of Calgary; male TRPA1^−/−^, B6;129P-Trpa1^tm1Kykw/J^, supplied by R. Yates, University of Calgary; female NOX2^−/−^, B6.129S-Cybb^tm1Din/J^, The Jackson Laboratories, West Grove, USA; male NOS1^−/−^, B6.129S4-Nos1^tm1Plh/J^, The Jackson Laboratories, West Grove, USA) (Figs. 2, D to F; 5A; 6, A to F; 7, E to H; 8; and 10, A and B and E to G; and figs. S1 and S4 to S6). The skin, ventral ribcage, diaphragm, lungs, heart, and all abdominal viscera except the kidneys were removed. The preparation was further reduced by rostral section to spare only thoracic segments. The g-SN was then tracked from the adrenal gland to the sympathetic chain. Last, the kidneys and remaining tissues below T13 were removed. The entire procedure was performed within 10 min. The preparation was then transferred to a recording chamber and secured ventral side up with 26-gauge needles on a Styrofoam base. The descending aorta was cannulated at both ends (T13 and at the aortic arch). The rostral cannula was connected to a pressure transducer, and the caudal cannula was supplied with perfusate via a peristaltic pump (Gilson Miniplus 3) controlled via a computerized pressure feedback system. Thus, the preparation was perfused via the descending aorta in the retrograde direction at constant pressure. The perfusate was equilibrated with 40-torr *P*CO_2_ balanced O_2_ (unless otherwise stated) and passed through a heat exchanger (34°C), bubble trap, and filter (25 μm) before reaching the preparation. The osmolarity of the perfusate was ~315 mosM, and the pH was ~7.4 (after equilibration). On initiating perfusion, the speed of the peristaltic pump was gradually increased to elevate the perfusion pressure to 90 mmHg. Perfusion pressure was then clamped. The perfusate leaking from the cut vessels in the preparation was recirculated after reoxygenation. Sympathetic activity was recorded from the g-SN via suction or hook electrode.

##### Pharmacological and gene KO in situ thoracic spinal cord experiments

Pharmacological and gene KO experiments to determine the SOS O_2_ sensing mechanism were performed in rat and mice preparations. All pharmacological protocols started with 5 min of baseline g-SN recordings, during which preparations received hyperoxic perfusate (570-torr *P*O_2_/40-torr *P*co_2_). Preparations then received an initial 5 min bout of mild (300-torr *P*O_2_/40-torr *P*co_2_) and/or severe (100-torr *P*O_2_/40-torr *P*co_2_) hypoxia, as indicated below. Following the first challenge and reperfusion with hyperoxic perfusate (5 min), nerve activity returned to baseline levels. Then, agonist(s) and/or antagonist(s) were added to the perfusate. Following the drug incubation period, which differed according to drug (see below), preparations were exposed to a second hypoxia challenge of the same level and duration as the first challenges. To eliminate the possibility that time and/or repetitive hypoxia challenges affect the hypoxic response, we performed time control experiments (Fig. 7E). In these experiments, preparations were exposed to two hypoxic stimuli (100-torr *P*O_2_/40-torr *P*CO_2_) separated by a period of 25 min of perfusion with hyperoxic aCSF. Drugs used were L-NNA (NOS1 antagonist, 15 nM, tested at 100-torr *P*O_2_ and incubated for 20 min; Fig. 7F) (*64*); l-arginine (NOS1 substrate, 100 μM, tested at 100-torr *P*O_2_ and incubated for 20 min; Fig. 7G) (*65*); SNP (NOS1-independent NO donor, 250 μM, tested at 100-torr *P*O_2_ and incubated for 20 min; Fig. 7H) (*66*); apocynin (NOX antagonist, 100 μM, tested at 100-torr *P*O_2_ and incubated for 20 min; Fig. 9A) (*67*); gadolinium (NOX agonist, 10 μM, tested at 100-torr *P*O_2_ and incubated for 20 min; Fig. 9B) (*38*); MnTMPyP (SOD mimetic, 25 μM, tested at 100-torr *P*O_2_ and incubated for 20 min; Fig. 9C) (*68*); hydrogen peroxide (H_2_O_2_, ROS, 100 μM, tested at 100-torr *P*O_2_ and incubated for 20 min; Fig. 9D) (*39*, *69*, *70*); AP-18 (TRPA1 antagonist, 10 μM, tested at 300- and 100-torr *P*O_2_ and incubated for 40 min; Fig. 10E) (*71*); HC030031 (TRPA1 antagonist, 30 μM, tested at 300- and 100-torr *P*O_2_ and incubated for 40 min; Fig. 10F) (*72*); 9-PNT (TRPM4 antagonist, 200 μM, tested at 100-torr *P*O_2_ and incubated for 20 min; Fig. 10G) (*73*); 2-APB (broadband TRP channel and IP3R blocker, 100 μM, tested at 100-torr *P*O_2_ and incubated for 20 min; fig. S5A) (*74*, *75*); or flufenamic acid (broadband TRP channel disrupter, 100 μM, tested at 100-torr *P*O_2_ and incubated for 20 min; fig. S5B) (*76*).

Wild-type and KO mice (NOS1^−/−^, NOX2^−/−^, and TRPA1^−/−^; Fig. 8) did not receive any drug treatment. Instead, g-SN responses were measured when tissue *P*O_2_ was ~100 torr (perfusate *P*O_2_, 300 torr) and during severe hypoxia (tissue *P*O_2_, ~6 torr; perfusate *P*O_2_, 100 torr).

For drug studies involving single levels of hypoxia, the predrug hypoxic response was assessed against the second hypoxic response in the presence of drug using paired *t* test. To determine whether a given drug affected g-SN activity before hypoxia, a one-sample *t* test was used. For studies involving two levels of hypoxia and/or KO mice, one-way or two-way ANOVAs were used. All data were tested for normality and/or difference/ratio consistency to inform the choice of test. Statistical analysis is presented in tables S2 and S3.

##### Hypercapnic response of in situ thoracic spinal cord

To test sensitivity to hypercapnia, the following protocol was used. Following a baseline recording under normoxia, preparations were exposed to a bout of hypoxia (100-torr *P*O_2_/40-torr *P*CO_2_ for 5 min; average tissue, 6.2 ± 10–torr *P*O_2_) and a bout of hypercapnia (570-torr *P*O_2_/60-torr *P*CO_2_ for 5 min). Perfusion *P*O_2_/*P*CO_2_ between bouts was returned to 570-torr *P*O_2_/40-torr *P*CO_2_ for 5 min.

#### Novel perfused in situ spinal cord, brainstem, and carotid body (triple perfused) preparation

This preparation was based on the dual perfused preparation in which brainstem and carotid bodies are perfused independently but added a third independently perfused compartment, the thoracic spinal cord (Figs. 3 and 4 and figs. S2 and S3). Brainstem and spinal cord perfusion was supplied via a double-lumen catheter, with one lumen used to record pressure. Pressure in both compartments was feedback controlled. The brainstem catheter entered via the left ventricle of the heart with the tip positioned and secured in the aortic arch. The spinal cord catheter was secured in the descending aorta for retrograde perfusion (with tip as caudal as possible ~L1 and aorta sutured closed at T3). The same perfusate as that used for in situ thoracic spinal cord preparations was used; perfusate was gassed, heated, passed through bubble traps, and filtered before entering the preparation. Unless otherwise stated, perfusion was equilibrated with gas mixtures containing 40-torr *P*CO_2_ balanced N_2_ (spinal cord and brainstem) or 35-torr *P*CO_2_ and 100-torr *P*O_2_ balanced O_2_ (carotid bodies). Separate dye experiments were performed to demonstrate that caudal and rostral sections of the neuronal axis were individually perfused (fig. S2). Briefly, the caudal and rostral compartments were perfused concurrently for 40 min with 0.05% Evans blue and 0.05% neutral red, respectively; the spinal cord and brainstem were then dissected and photographed, and an ImageJ macro incorporating the isodata threshold routine was used to automatically determine the dye boundary. With the descending aorta ligated at T3, rostral spill overextended to T4. Consequently, using this preparation is likely to overestimate the importance of the brainstem and underestimate the importance of the thoracic spinal cord in oxygen sensing because the brainstem compartment includes several rostral thoracic segments. Hook and suction electrodes were used to record from the right phrenic and right or left sympathetic nerves.

#### Data analysis for in situ preparations

Data were analyzed offline. Phrenic and sympathetic nerve recordings were analyzed using in-house automated software written by R.J.A.W. Raw splanchnic nerve activity was divided into 10-s bins, and neural activity in each bin was rectified and summed (expressed as integrated neural response) or spike-counted with a threshold set at 4 SDs of the signal immediately before the first challenge. Integrated phrenic activity was analyzed to determine burst amplitude, frequency, and eupneic index, where 0 is a rapid onset decrementing (gasp-like) burst and 1 is an augmenting rapid offset (eupneic-like) burst.

Unless otherwise stated, we defined baseline as activity preceding one or consecutive hypoxia bouts over at least a minute. For drug effects alone, the baseline was measured immediately preceding drug onset. Neural responses during hypoxia or drug were normalized to baseline.

### En bloc transverse thoracic spinal cord section preparation

Wistar and Sprague-Dawley rats of either sex (0 to 4 days old) were used for these experiments (Figs. 5, B and C; 6G; and 10, H to J). The basic methods have been described previously (*77*). Briefly, animals were heavily anesthetized with isoflurane such that the foot pinch reflex was lost and then decapitated. The spinal cord was excised, and transverse sections (including two segments of the second to six thoracic spinal cord, Th2 to Th6) were resected with microscissors and placed in aCSF (25° to 26°C). The aCSF was composed of 118 mM NaCl, 26 mM NaHCO_3_, 3.0 mM KCl, 1.0 mM MgCl_2_, 1.0 mM CaCl_2_, 1.2 mM NaH_2_PO_4_, and 30 mM d-glucose (pH 7.4). Unless otherwise stated, perfusate was gassed with 95% O_2_/5% CO_2_. Preparations were continuously superfused with aCSF at a rate of 15 ml/min (Fig. 5B; Calgary) or 3 ml/min (Figs. 5C, 6G, and 10, H to J; Japan) depending on the recording chamber.

#### En bloc section tissue PO_2_

To measure tissue *P*O_2_, we used Clark-style *P*O_2_ microelectrodes as described above, while recording ventral root nerve activity (Fig. 5B). Electrodes were calibrated before and after each set of experiments in saline of the same composition used for superfusion. The electrode was lowered until the tip touched the surface of the section, identified upon loss of *P*O_2_ fluctuations associated with perfusate turbulence. The electrode tip was then lowered into the tissue to a depth of 100 μm. Once a stable tissue *P*O_2_ was obtained, we decreased the gas concentration used to equilibrate the perfusate from 570- to 0-torr *P*O_2_ in a single step. Approximately 2 min after the gas change (i.e., time to equilibrate the perfusate and transport the perfusate to the rig), we observed an exponential decay in tissue *P*O_2_ toward 0 torr. After 5 min, the *P*O_2_ used to equilibrate the perfusate was returned to 570 torr. Responders (i.e., preparations that mounting a spike frequency response of >4 times SD of baseline) and no responders were identified using automated software. The onset duration and *P*O_2_ of responses as defined above were also determined.

#### En bloc section whole-cell patch

The experiments were performed in Tokyo at sea level (gases were not adjusted for daily changes in atmospheric pressure and are reported in percentage) (Figs. 5C and 10, H to J). Intracellular recordings of IML neurons were made in the en bloc transverse thoracic spinal cord section preparations, using the whole-cell patch-clamp technique. Patch micropipettes were filled with 130 mM K-gluconate, 1 mM MgCl_2_, 10 mM Hepes, 10 mM EGTA, 1 mM CaCl_2_, and 2 mM Na_2_-ATP, adjusted to a pH 7.3 with KOH. For histological analysis of the recorded cells, electrode tips were filled with 0.5% Lucifer yellow (lithium salt, Sigma-Aldrich, Japan). SSPNs in the IML were identified by their all-or-none antidromic responses to ventral root stimulation applied with a suction electrode (1 to 10 V; duration, 100 μs). Four neurons that passed the above test were excluded from data analysis because subsequent histological analysis demonstrated them to be outside the IML (ventral motor nucleus and medial to the IML).

After the electrophysiological confirmation of SSPN’s by backfiring the ventral root, some preparations were incubated in either TTX (0.5 μM) only (control) or TTX combined with one of the following (Figs. 5C and 10, G to I): HC030031 (10 μM) or 9-PNT (200 μM). After the incubation period (10 to 30 min) during which the section was superfused with aCSF equilibrated with 95% O_2_/5% CO_2_, the superfusate was switched to hypoxic aCSF equilibrated with 0% O_2_/5% CO_2_ for 5 to 8 min. Fractional concentrations (F) of O_2_ in the dish were assessed retroactively under the same conditions using a Clark-style polarographic electrode. Dish FO_2_ during control conditions was 87%; the nadir during the challenge was 13%. Statistical analysis of these data is summarized in table S4. In some preparations, we observed increased high-frequency fluctuations in membrane potential (flickering; see Fig. 10J, inlay) with hypoxia. Quantification using custom software demonstrates that HC030031 or 9-PNT abolishes hypoxia-induced flickering (Fig. 10J). Cell input resistance was monitored by applying square current pulses (500 ms, 10 to 80 pA) every 10 s. After 5 to 8 min of hypoxic stimulation, the perfusate was returned to 95% O_2_/5% CO_2_ solution in the presence of TTX. A total of 10 positively identified SSPNs were examined.

#### En bloc calcium imaging of astrocytes

Experiments were performed in en bloc transverse thoracic spinal cord section preparations of neonatal Wistar rats (P0 to P4, male and female) around thoracic levels 2 and 3 (Fig. 6G). A glass micropipette was used to pressure-inject 200 μM Oregon Green 488 BAPTA-1 AM (Invitrogen, Carlsbad, CA) in the proximity of the IML and preparations left to incubate for 20 min while being continuously superfused. This dye stains both astrocytes and neurons (*77*). The cell-bound dye in the IML was excited at 488 nm using a laser diode (Cobolt 06-MLD, HÜBNER Photonics, Kassel, Germany), and cellular activities were visualized through a 520-nm long-pass emission filter. Images were captured every 0.3 s using a Nipkow-disk confocal scanner unit (CSU21, Yokogawa Electric, Tokyo, Japan), an electron multiplying charge-coupled device camera (Luca S 658 M, Andor Technology, Belfast, Northern Ireland, UK), an upright fluorescent microscope (Eclipse E600FN, Nikon, Tokyo, Japan), and a water-immersion objective lens (40×, 0.8 numerical aperture, Fluor, Nikon) (*78*). Two sets of experiments were performed: one perfused with control (*n* = 5) and the other with 250 μM FCA (*n* = 6). Care was taken in preparing FCA to remove barium (*34*). In control experiments, preparations were imaged for 120 s. Then, to classify recorded cells into astrocytes and neurons, preparations were perfused with 0.2 mM K^+^ (low K^+^) for 180 s, which exclusively activates astrocytes (*79*). Imaging was then paused and perfusion with normal saline resumed. Twenty-four minutes after washout, preparations were again imaged. After 60 s, preparations were perfused with 0% O_2_ for 240 s. One control preparation was incubated with TTX; cellular responses to low K^+^ persisted in this preparation. In FCA-treated preparations, preparations were preincubated with FCA for 27 min before imaging. FCA-treated preparations were then imaged for 60 s and challenged with 0% O_2_ for 240 s. After the challenge, imaging was paused, and the solution was returned to 95% O_2_/5% CO_2_. Twenty-four minutes after washout, imaging was resumed, and after 120 s, preparations were perfused with low K^+^ for a further 180 s. Analysis was performed using automated software written by R.J.A.W. Briefly, frames were stacked, registered, and filtered using a three-dimensional median filter. The first 50 frames were removed because of a light-intensity settling artifact and the remaining stack corrected for bleaching using an exponential fit; the stack was further processed to remove background. Mean (see images in Fig. 6G) and SD *Z*-projection images were generated. The SD *Z*-projection was median-filtered, and the noise (defined as mean plus 5× SD) was subtracted; the resulting image was filtered to enhance remaining differences. The ImageJ local maxima algorithm was then used to define regions of interest (ROIs) (stained cells) in the processed SD *Z*-projection. ROIs were then used to process stacks, extracting changes in cell intensity (i) with time. To identify responders, time series were differentiated and selected if the maximum *di*/*dt* during the protocol exceeds the max + peak *di*/*dt* of the baseline. Responders were then normalized to baseline and overlaid.

### Isolated perfused carotid body preparation

To compare O_2_ sensitivity of the spinal cord and the carotid body, we used the artificially perfused carotid body (fig. S1). We constructed an oxygen dose-response curve for the carotid body and plotted with the dose-response curve for the artificially perfused thoracic in situ spinal cord preparation. The carotid body preparation has been described elsewhere (*80*, *81*). Please note that the carotid body is densely irrigated with blood vessels that are thought to minimize tissue O_2_ gradients and thereby accounting for its ability to respond to *P*O_2_, not SO_2_. As the carotid body *P*O_2_-response curve of the artificially perfused carotid body preparation is similar to that of carotid body afferents measured in vivo (*82*), we assume that tissue *P*O_2_ in this preparation is similar to that of the perfusate. To generate a hypoxic dose-response curve for the carotid body, we used a similar protocol as used for the in situ spinal cord preparation (Fig. 2D). To aid comparison, relative change in activity of both preparations was determined and normalized to that when both tissues were assumed to be at 100-torr *P*O_2_.

### Histochemical cell labeling

#### c-fos/ChAT immunohistochemistry

All *c-fos* expression experiments were performed using the in situ thoracic spinal cord preparation from male Sprague-Dawley P15 rats (Figs. 6, A and B, and 10B). TTX (1 μM) was used in experiments shown in Fig. 6 (A and B) to block network transmission and thereby isolate the primary oxygen-sensitive neurons. Perfusate containing high Mg^2+^/zero Ca^2+^ was used in experiments shown in Fig. 10B to demonstrate the necessity of calcium in SOS neuronal responses to hypoxia. After the standardized reperfusion protocol (see above), we waited for 60 min and then exposed the spinal cord to three bouts of hypoxia (60-torr *P*O_2_/40-torr *P*CO_2_/balanced N_2_). Each bout lasted for 5 min, with 5-min intervals between bouts. After the final hypoxic challenge, the spinal cord was perfused for another 60 min with perfusate equilibrated with 570-torr *P*O_2_/40-torr *P*CO_2_. The preparation was then removed from the recording chamber and immediately immersed in paraformaldehyde. For control experiments, we perfused spinal cord preparations with TTX or high Mg^2+^/zero Ca^2+^ solution for the same duration but without hypoxic exposures.

Following hypoxic challenges, preparations were paraformaldehyde-fixed via immersion for 24 hours in 50 ml of 4% paraformaldehyde (Sigma-Aldrich, St. Louis, MO) diluted in 0.1 M phosphate buffer (PB) (pH 7.4) at 4°C. The spinal cords were dissected out and cryoprotected in 30% sucrose dissolved in 0.1 M PB (pH 7.4) for 24 hours at 4°C. Subsequently, tissues were frozen to −20°C for sectioning. Free-floating sections were cut using a cryostat (Leica CM1850, Wetzlar, Germany) at a thickness of 30 μm.

Sections were dipped in 0.1 M PB and washed with 0.03% H_2_O_2_ in 0.1 M PB for 20 min, followed by 0.1 M PB alone (3× for 10 min) and 0.05 M tris-buffered saline (TBS) containing 0.25% Triton X-100 (TBS-Tx; 1× for 10 min). Sections were further incubated in 2% normal donkey serum (NDS; S30, Merck Millipore, Billerca, MA, USA) diluted in 0.05 M TBS-Tx (40 min) at room temperature (RT) and then in rabbit anti-*c-fos* antibody for 48 hours at 4°C (1:10,000; catalog no. ABE457, Merck Millipore, Billerca, MA, USA) diluted in 0.05 M TBS-Tx. Briefly, sections were washed in 0.05 M TBS-Tx (3× for 10 min) and incubated in biotinylated donkey anti-rabbit conjugate (catalog no. 711-065-152, Jackson ImmunoResearch Laboratories, West Grove, PA, USA) diluted at 1:200 in 0.05 M TBS-Tx for 2 hours at RT. Sections were then washed three times (10 min each) in 0.05 M TBS-Tx and incubated in avidin-biotin complex (ABC kit, catalog no. PK-6100, VECTASTAIN, Vector Labs) diluted at 1:400 in 0.05 M TBS-Tx (2 hours at RT). Subsequently, sections were washed in 0.05 M Tris-HCl (3× for 10 min) and incubated in DAB substrate containing nickel and peroxide buffer (catalog no. SK-4100, Vector Labs) for 10 min to produce a black staining. Sections were then washed in 0.05 M Tris-HCl (3× 10 min) and further incubated in goat anti-ChAT antibody (1:1000; catalog no. AB144P, Merck Millipore, Billerca, MA, USA) diluted in 0.05 M TBS-Tx for 48 hours at 4°C. Sections were then washed in 0.05 M TBS-Tx (3× for 10 min) and incubated in biotinylated donkey anti-goat conjugate (catalog no. 705-067-003, Jackson ImmunoResearch Laboratories, West Grove, PA, USA) diluted at 1:200 in 0.05 M TBS-Tx for 2 hours at RT. Sections were then washed three times (10 min each) in 0.05 M TBS-Tx and incubated in ABC kit diluted at 1:400 in 0.05 M TBS-Tx (2 hours at RT), followed by three further washes in 0.05 M tris (10 min each) and incubation in DAB substrate and peroxide buffer for 10 min to produce a brown staining. Sections were then washed in 0.05 M Tris-HCl (3× for 10 min) and mounted onto slides coated with 0.4% gelatin (Sigma-Aldrich) and coverslipped using Permount (Electron Microscopy Sciences).

##### Cell quantification

The cell quantification was performed using a double-blind study design. For cell counting, we sampled intermediate (T5 to T9) segments of the thoracic spinal cord. The number of *c-fos*–immunoreactive (*c-fos*–ir) cell nuclei in each segment was sampled from 10 30-μm sections randomly selected from hypoxic (*n* = 6) and hyperoxic (time control; *n* = 6) experimental groups. *c-fos*–ir nuclei were counted in randomly selected sections in ChAT^+^ neurons only. In each preparation, we calculated the average number of *c-fos*–ir cells per section.

#### NOS1 (neuronal NOS) immunohistochemistry

To confirm NOS1 expression in the IML, we used spinal cords from adult male Sprague-Dawley rats. Animals were anesthetized, and the thoracic segments were isolated as per the perfused in situ thoracic spinal cord preparation (Fig. 7A). The fixation, cryoprotection, and sectioning were performed as previously described for the *c-fos* and ChAT double immunohistochemistry. After sectioning, spinal cord sections were dipped in 0.1 M PB and washed with 0.03% H_2_O_2_ in 0.1 M PB for 20 min, followed by 0.1 M PB alone (3× in 10 min) and 0.05 M TBS-Tx (1× for 10 min). Sections were further incubated in 2% NDS diluted in 0.05 M TBS-Tx (40 min at RT) and then in a rabbit anti–neuronal NOS (nNOS) antibody (1:6000; catalog no. ab229785, Abcam, Toronto, Canada) diluted in 0.05 M TBS-Tx for 48 hours at 4°C. Briefly, sections were washed in 0.05 M TBS-Tx (3× for 10 min), incubated in a biotinylated donkey anti-rabbit conjugate (catalog no. 711-065-152, Jackson ImmunoResearch Laboratories, West Grove, PA, USA) diluted at 1:200 in 0.05 M TBS-Tx for 2 hours at RT. Sections were then washed three times (10 min each) in 0.05 M TBS-Tx and incubated in an ABC kit diluted at 1:400 in 0.05 M TBS-Tx (2 hours at RT), followed by three further washes in 0.05 M tris (10 min each) and incubation in DAB substrate nickel concentrate and peroxide buffer for 10 min to produce a black staining. Sections were then washed in 0.05 M Tris-HCl (3× for 10 min) and mounted onto slides coated with 0.4% gelatin (Sigma-Aldrich) and coverslipped using Permount (Electron Microscopy Sciences).

#### NOS1/ChAT fluorescent immunohistochemistry

The fixation, cryoprotection, and sectioning of mouse spinal cord were performed as previously described for rat spinal cords (Fig. 7, B to D). After sectioning, adult C57Bl/6 mice spinal cord sections were dipped in 0.1 M PB and washed with 0.05 M TBS-Tx (1× for 10 min). Sections were further incubated in 2% NDS diluted in 0.05 M TBS-Tx (40 min at RT) and then in rabbit anti-nNOS antibody (1:6000; catalog no. ab229785, Abcam, Toronto, Canada) and goat anti-ChAT antibody (1:1000; catalog no. ab144P, Merck Millipore, Billerca, MA, USA) diluted in 0.05 M TBS-Tx for 48 hours at 4°C. Briefly, sections were washed in 0.05 M TBS-Tx (3x for 10 min) and further incubated in Alexa Fluor 594–conjugated donkey anti-rabbit (1:400; catalog no. A-21207, Thermo Fisher Scientific, Waltham, MA, USA) and Alexa Fluor 488–conjugated donkey anti-goat (1:400; catalog no. A-11055, Thermo Fisher Scientific, Waltham, MA, USA) in 0.05 M TBS-Tx for 2 hours at RT. Sections were then washed in 0.05 M Tris-HCl (3× for 10 min), mounted onto slides coated with 0.1% gelatin (Sigma-Aldrich), and coverslipped using Fluoromount (H-1000, Vector Labs).

#### Image acquisition for immunohistochemistry

After histological processing of *c-fos*/ChAT and NOS1 immunohistochemistry, sections were examined, and images were acquired using a Zeiss Axioplan 2 microscope (Zeiss, Germany), equipped with a digital camera (Zeiss AxioCam MRc). For confocal analysis of the ChAT/NOS1 double fluorescent immunohistochemistry, a multiphoton confocal microscope (A1 MP, Nikon, Tokyo, Japan) was used. A 499-nm diode laser was used to excite Alexa Fluor 488, and images were captured at the emission band peak of 520 nm. For the detection of Alexa Fluor 594, a 590-nm diode laser was used, and images were captured at the emission band peak of 618 nm. *Z*-stack images were captured with multiple images, each image was taken with stepwise depth of 0.8 to 1.0 μm in the *z* plane. Digital images from the microscopy were full-field modified to optimize for image resolution, brightness, and contrast (Adobe Photoshop CS5 extended, version 12.0; Adobe Systems Inc., San Jose, CA), so as to best represent the immunohistochemistry observed during microscope analysis.

#### ISH histochemistry

To determine the expression of TRPA1 in the spinal cord, five Wistar rat neonates (Japan SLC, Shizuoka, Japan; P2, *n* = 3) and juvenile (P22, *n* = 2) were deeply anesthetized with ether and decapitated (Fig. 10C). ISH was performed on spinal cords (T1 to T6) and dorsal root ganglia. Dorsal root ganglia and spinal cords were fixed for 2 to 3 hours and 3 to 5 hours, respectively, at 4°C in 4% paraformaldehyde in 0.1 M phosphate-buffered saline. Samples were immersed in 18% sucrose/Hank’s balanced salt solution, embedded in optimal cutting temperature compound (Sakura Finetek, Torrance, CA), then frozen on dry ice, and cut into 30-μm-thick cryosections. A digoxigenin–uridine 5′-triphosphate (Roche Diagnostics, Basel, Switzerland)–labeled riboprobe for rat Trpa1 was used (*83*). Images of samples were obtained with ×10 or ×20 magnification on a conventional fluorescence microscope (BX51, Olympus, Tokyo, Japan).

### Additional statistical analysis

Additional statistical analysis can be found in tables S1 to S4.

## Notes

View/request a protocol for this paper from *Bio-protocol*.

## References

[1] P. Kumar, N. R. Prabhakar, Peripheral chemoreceptors: Function and plasticity of the carotid body. Compr.Physiol. 2, 141–219 (2012). 23728973 10.1002/cphy.c100069 PMC3919066

[2] P. Ortega-Sáenz, J. López-Barneo, Physiology of the carotid body: From molecules to disease. Annu. Rev. Physiol. 82, 127–149 (2020). 31618601 10.1146/annurev-physiol-020518-114427

[3] A. V. Gourine, G. D. Funk, On the existence of a central respiratory oxygen sensor. J. Appl. Physiol. 123, 1344–1349 (2017). 28522760 10.1152/japplphysiol.00194.2017 PMC5792097

[4] T. Bernthal, C. C. Woodcock, Responses of the vasomotor center to hypoxia after denervation of carotid and aortic bodies. Am. J. Physiol. 166, 45–54 (1951). 14857147 10.1152/ajplegacy.1951.166.1.45

[5] M. J. Wasicko, J. E. Melton, J. A. Neubauer, N. Krawciw, N. H. Edelman, Cervical sympathetic and phrenic nerve responses to progressive brain hypoxia. J. Appl. Physiol. 68, 53–58 (1990). 2107169 10.1152/jappl.1990.68.1.53

[6] C. G. Morrill, J. R. Meyer, J. V. Weil, Hypoxic ventilatory depression in dogs. J. Appl. Physiol. 38, 143–146 (1975). 1110230 10.1152/jappl.1975.38.1.143

[7] M. K. Sun, D. J. Reis, Central neural mechanisms mediating excitation of sympathetic neurons by hypoxia. Prog. Neurobiol. 44, 197–219 (1994). 7831477 10.1016/0301-0082(94)90038-8

[8] E. M. Horn, T. G. Waldrop, Oxygen-sensing neurons in the caudal hypothalamus and their role in cardiorespiratory control. Respir. Physiol. 110, 219–228 (1997). 9407614 10.1016/s0034-5687(97)00086-8

[9] O. Pascual, M.-P. Morin-Surun, B. Barna, M. Denavit-Saubié, J.-M. Pequignot, J. Champagnat, Progesterone reverses the neuronal responses to hypoxia in rat nucleus tractus solitarius in vitro. J. Physiol. 544, 511–520 (2002). 12381823 10.1113/jphysiol.2002.023994 PMC2290600

[10] J. A. Neubauer, J. Sunderram, Oxygen-sensing neurons in the central nervous system. J. Appl. Physiol. 96, 367–374 (2004). 14660498 10.1152/japplphysiol.00831.2003

[11] P. G. Guyenet, Regulation of breathing and autonomic outflows by chemoreceptors. Compr. Physiol. 4, 1511–1562 (2014). 25428853 10.1002/cphy.c140004 PMC4794276

[12] A. K. Curran, J. R. Rodman, P. R. Eastwood, K. S. Henderson, J. A. Dempsey, C. A. Smith, Ventilatory responses to specific CNS hypoxia in sleeping dogs. J. Appl. Physiol. 88, 1840–1852 (2000). 10797149 10.1152/jappl.2000.88.5.1840

[13] A. M. Evans, A. D. Mahmoud, J. Moral-Sanz, S. Hartmann, The emerging role of AMPK in the regulation of breathing and oxygen supply. Biochem. J. 473, 2561–2572 (2016). 27574022 10.1042/BCJ20160002 PMC5003690

[14] J. Sunderram, J. Semmlow, P. Patel, H. Rao, G. Chun, P. Agarwala, M. Bhaumik, O. Le-Hoang, S.-E. Lu, J. A. Neubauer, Heme oxygenase-1-dependent central cardiorespiratory adaptations to chronic intermittent hypoxia in mice. J. Appl. Physiol. 121, 944–952 (2016). 27609199 10.1152/japplphysiol.00036.2016

[15] E. M. Horn, T. G. Waldrop, Hypoxic augmentation of fast-inactivating and persistent sodium currents in rat caudal hypothalamic neurons. J. Neurophysiol. 84, 2572–2581 (2000). 11067999 10.1152/jn.2000.84.5.2572

[16] R. Kaya, E. H. Starling, Note on asphyxia in the spinal animal. J. Physiol. 39, 346–353 (1909). 16992986 10.1113/jphysiol.1909.sp001341 PMC1533663

[17] C. V. Rohlicek, C. Polosa, Hypoxic responses of sympathetic preganglionic neurons in the acute spinal cat. Am. J. Phys. 241, H679–H683 (1981). 10.1152/ajpheart.1981.241.5.H679 7304756

[18] J. H. Coote, The organisation of cardiovascular neurons in the spinal cord. Rev. Physiol. Biochem. Pharmacol. 110, 147–285 (1988). 3285441 10.1007/BFb0027531

[19] R. S. Alexander, The effects of blood flow and anoxia on spinal cardiovascular centers. Am. J. Physiol. Legacy Content. 143, 698–708 (1945).

[20] G. C. Mathison, The action of asphyxia upon the spinal animal. J. Physiol. 41, 416–449 (1910). 16993035 10.1113/jphysiol.1910.sp001411 PMC1512900

[21] V. Rajani, Y. Zhang, V. Jalubula, V. Rancic, S. SheikhBahaei, J. D. Zwicker, S. Pagliardini, C. T. Dickson, K. Ballanyi, S. Kasparov, A. V. Gourine, G. D. Funk, Release of ATP by pre-Bötzinger complex astrocytes contributes to the hypoxic ventilatory response via a Ca^2+^-dependent P2Y_1_ receptor mechanism. J. Physiol. (Lond.) 596, 3245–3269 (2018). 28678385 10.1113/JP274727 PMC6068109

[22] J. M. Ramirez, L. J. Severs, S. C. Ramirez, I. M. Agosto-Marlin, Advances in cellular and integrative control of oxygen homeostasis within the central nervous system. J. Physiol. 596, 3043–3065 (2018). 29742297 10.1113/JP275890 PMC6068258

[23] M. Uchiyama, A. Nakao, Y. Kurita, I. Fukushi, K. Takeda, T. Numata, H. N. Tran, S. Sawamura, M. Ebert, T. Kurokawa, R. Sakaguchi, A. J. Stokes, N. Takahashi, Y. Okada, Y. Mori, O_2_-dependent protein internalization underlies astrocytic sensing of acute hypoxia by restricting multimodal TRPA1 channel responses. Curr. Biol. 30, 3378–3396.e7 (2020). 32679097 10.1016/j.cub.2020.06.047

[24] H. N. Sapru, E. R. Gonzalez, A. J. Krieger, Greater splanchnic nerve activity in the rat. Brain Res. Bull. 8, 267–272 (1982). 7093734 10.1016/0361-9230(82)90058-2

[25] E. Ortiz-Prado, J. F. Dunn, J. Vasconez, D. Castillo, G. Viscor, Partial pressure of oxygen in the human body: A general review. Am. J. Blood Res. 9, 1–14 (2019). 30899601 PMC6420699

[26] P. R. Angelova, V. Kasymov, I. Christie, S. Sheikhbahaei, E. Turovsky, N. Marina, A. Korsak, J. Zwicker, A. G. Teschemacher, G. L. Ackland, G. D. Funk, S. Kasparov, A. Y. Abramov, A. V. Gourine, Functional oxygen sensitivity of astrocytes. J. Neurosci. 35, 10460–10473 (2015). 26203141 10.1523/JNEUROSCI.0045-15.2015 PMC4510287

[27] D. G. S. Farmer, N. Pracejus, B. Dempsey, A. Turner, P. Bokiniec, J. F. R. Paton, A. E. Pickering, J. Burguet, P. Andrey, A. K. Goodchild, R. M. McAllen, S. McMullan, On the presence and functional significance of sympathetic premotor neurons with collateralized spinal axons in the rat. J. Physiol. 597, 3407–3423 (2019). 31077360 10.1113/JP277661

[28] A. F. DiMarco, K. E. Kowalski, High-frequency spinal cord stimulation of inspiratory muscles in dogs: A new method of inspiratory muscle pacing. J. Appl. Physiol. 107, 662–669 (2009). 19520839 10.1152/japplphysiol.00252.2009 PMC4073921

[29] M. Rusnak, Z. E. Tóth, S. B. House, H. Gainer, Depolarization and neurotransmitter regulation of vasopressin gene expression in the rat suprachiasmatic nucleus in vitro. J. Neurosci. 27, 141–151 (2007). 17202481 10.1523/JNEUROSCI.3739-06.2007 PMC6672276

[30] A. V. Gourine, V. Kasymov, N. Marina, F. Tang, M. F. Figueiredo, S. Lane, A. G. Teschemacher, K. M. Spyer, K. Deisseroth, S. Kasparov, Astrocytes control breathing through pH-dependent release of ATP. Science 329, 571–575 (2010). 20647426 10.1126/science.1190721 PMC3160742

[31] F. Tang, S. Lane, A. Korsak, J. F. R. Paton, A. V. Gourine, S. Kasparov, A. G. Teschemacher, Lactate-mediated glia-neuronal signalling in the mammalian brain. Nat. Commun. 5, 3284 (2014). 24518663 10.1038/ncomms4284 PMC3926012

[32] G. R. J. Gordon, H. B. Choi, R. L. Rungta, G. C. R. Ellis-Davies, B. A. MacVicar, Brain metabolism dictates the polarity of astrocyte control over arterioles. Nature 456, 745–749 (2008). 18971930 10.1038/nature07525 PMC4097022

[33] W. Shen, L. Nikolic, C. Meunier, F. Pfrieger, E. Audinat, An autocrine purinergic signaling controls astrocyte-induced neuronal excitation. Sci. Rep. 7, 11280 (2017). 28900295 10.1038/s41598-017-11793-x PMC5595839

[34] J. L. Stringer, K. Mukherjee, T. Xiang, K. Xu, Regulation of extracellular calcium in the hippocampus in vivo during epileptiform activity—Role of astrocytes. Epilepsy Res. 74, 155–162 (2007). 17434291 10.1016/j.eplepsyres.2007.03.005 PMC1945156

[35] U. Förstermann, W. C. Sessa, Nitric oxide synthases: Regulation and function. Eur. Heart J. 33, 829–837 (2012). 21890489 10.1093/eurheartj/ehr304 PMC3345541

[36] R. P. Brandes, N. Weissmann, K. Schröder, Nox family NADPH oxidases: Molecular mechanisms of activation. Free Radic. Biol. Med. 76, 208–226 (2014). 25157786 10.1016/j.freeradbiomed.2014.07.046

[37] D. Blottner, H. G. Baumgarten, L-NNA inhibits the histochemical NADPH-d reaction in rat spinal cord neurons. Histochem. Cell Biol. 103, 379–385 (1995). 7641070 10.1007/BF01457813

[38] B. Wagner, V. Drel, Y. Gorin, Pathophysiology of gadolinium-associated systemic fibrosis. Am. J. Physiol. Renal Physiol. 311, F1–F11 (2016). 27147669 10.1152/ajprenal.00166.2016 PMC4967166

[39] E. Leiva-Salcedo, D. Riquelme, O. Cerda, A. Stutzin, TRPM4 activation by chemically- and oxygen deprivation-induced ischemia and reperfusion triggers neuronal death. Channels (Austin) 11, 624–635 (2017). 28876976 10.1080/19336950.2017.1375072 PMC5786181

[40] K. Panda, S. Adak, D. Konas, M. Sharma, D. J. Stuehr, A conserved aspartate (Asp-1393) regulates NADPH reduction of neuronal nitric-oxide synthase implications for catalysis. J. Biol. Chem. 279, 18323–18333 (2004). 14966111 10.1074/jbc.M310391200

[41] G. J. Gatto Jr., Z. Ao, M. G. Kearse, M. Zhou, C. R. Morales, E. Daniels, B. T. Bradley, M. T. Goserud, K. B. Goodman, S. A. Douglas, M. R. Harpel, D. G. Johns, NADPH oxidase-dependent and -independent mechanisms of reported inhibitors of reactive oxygen generation. J. Enzyme Inhib. Med. Chem. 28, 95–104 (2013). 22136506 10.3109/14756366.2011.636360

[42] G. L. Semenza, N. R. Prabhakar, The role of hypoxia-inducible factors in carotid body (patho) physiology. J. Physiol. 596, 2977–2983 (2018). 29359806 10.1113/JP275696 PMC6068252

[43] A. R. Cross, L. Henderson, O. T. Jones, M. A. Delpiano, J. Hentschel, H. Acker, Involvement of an NAD(P)H oxidase as a pO2 sensor protein in the rat carotid body. Biochem. J. 272, 743–747 (1990). 2268299 10.1042/bj2720743 PMC1149771

[44] R. J. Rakoczy, C. N. Wyatt, Acute oxygen sensing by the carotid body: A rattlebag of molecular mechanisms. J. Physiol. 596, 2969–2976 (2018). 29214644 10.1113/JP274351 PMC6068253

[45] C. T. Migita, K. M. Matera, M. Ikeda-Saito, J. S. Olson, H. Fujii, T. Yoshimura, H. Zhou, T. Yoshida, The oxygen and carbon monoxide reactions of heme oxygenase. J. Biol. Chem. 273, 945–949 (1998). 9422754 10.1074/jbc.273.2.945

[46] H. L. Bonkovsky, J. F. Healey, J. Pohl, Purification and characterization of heme oxygenase from chick liver. Comparison of the avian and mammalian enzymes. Eur. J. Biochem. 189, 155–166 (1990). 2158889 10.1111/j.1432-1033.1990.tb15472.x

[47] C.-K. Su, Y.-Y. Chen, C.-M. Ho, Nitric oxide orchestrates a power-law modulation of sympathetic firing behaviors in neonatal rat spinal cords. Front. Physiol. 9, 163 (2018). 29559921 10.3389/fphys.2018.00163 PMC5845561

[48] P. MacFarlane, S. Vinit, G. Mitchell, Spinal nNOS regulates phrenic motor facilitation by a 5-HT2B receptor- and NADPH oxidase-dependent mechanism. Neuroscience 269, 67–78 (2014). 24680940 10.1016/j.neuroscience.2014.03.014 PMC4361021

[49] H. Vais, A. P. Siebert, Z. Ma, M. Fernández-Mongil, J. K. Foskett, D.-O. D. Mak, Redox-regulated heterogeneous thresholds for ligand recruitment among InsP3R Ca^2+^-release channels. Biophys. J. 99, 407–416 (2010). 20643058 10.1016/j.bpj.2010.04.034 PMC2905128

[50] N. Takahashi, T. Kuwaki, S. Kiyonaka, T. Numata, D. Kozai, Y. Mizuno, S. Yamamoto, S. Naito, E. Knevels, P. Carmeliet, T. Oga, S. Kaneko, S. Suga, T. Nokami, J. Yoshida, Y. Mori, TRPA1 underlies a sensing mechanism for O_2_. Nat. Chem. Biol. 7, 701–711 (2011). 21873995 10.1038/nchembio.640

[51] M. L. Marcus, D. D. Heistad, J. C. Ehrhardt, F. M. Abboud, Regulation of total and regional spinal cord blood flow. Circ. Res. 41, 128–134 (1977). 862136 10.1161/01.res.41.1.128

[52] R. Hickey, M. S. Albin, L. Bunegin, J. Gelineau, Autoregulation of spinal cord blood flow: Is the cord a microcosm of the brain? Stroke 17, 1183–1189 (1986). 3810718 10.1161/01.str.17.6.1183

[53] R. J. A. Wilson, L. J. Teppema, Integration of central and peripheral respiratory chemoreflexes. Compr. Physiol. 6, 1005–1041 (2016). 27065173 10.1002/cphy.c140040

[54] M. K. Sun, D. J. Reis, Hypoxic excitation of medullary vasomotor neurons in rats are not mediated by glutamate or nitric oxide. Neurosci. Lett. 157, 219–222 (1993). 7694196 10.1016/0304-3940(93)90741-3

[55] A. Moreno-Domínguez, P. Ortega-Sáenz, L. Gao, O. Colinas, P. García-Flores, V. Bonilla-Henao, J. Aragonés, M. Hüttemann, L. I. Grossman, N. Weissmann, N. Sommer, J. López-Barneo, Acute O_2_ sensing through HIF2α-dependent expression of atypical cytochrome oxidase subunits in arterial chemoreceptors. Sci. Signal. 13, eaay9452 (2020). 31848220 10.1126/scisignal.aay9452

[56] I. Kim, L. Fite, D. F. Donnelly, J. H. Kim, J. L. Carroll, Possible role of TRP channels in rat glomus cells. Adv. Exp. Med. Biol. 860, 227–232 (2015). 26303485 10.1007/978-3-319-18440-1_25

[57] A. Roy, M. M. J. Farnham, F. Derakhshan, P. M. Pilowsky, R. J. A. Wilson, Acute intermittent hypoxia with concurrent hypercapnia evokes P2X and TRPV1 receptor-dependent sensory long-term facilitation in naïve carotid bodies. J. Physiol. 596, 3149–3169 (2018). 29159869 10.1113/JP275001 PMC6068228

[58] H. H. Lin, C.-H. Chen, W.-K. Hsieh, T. H. Chiu, C.-C. Lai, Hydrogen peroxide increases the activity of rat sympathetic preganglionic neurons in vivo and in vitro. Neuroscience 121, 641–647 (2003). 14568024 10.1016/s0306-4522(03)00517-7

[59] V. A. Braga, J. F. R. Paton, B. H. Machado, Ischaemia-induced sympathoexcitation in spinalyzed rats. Neurosci. Lett. 415, 73–76 (2007). 17254709 10.1016/j.neulet.2006.12.045

[60] E. C. Hart, Human hypertension, sympathetic activity and the selfish brain. Exp. Physiol. 101, 1451–1462 (2016). 27519960 10.1113/EP085775

[61] G. S. Mitchell, M. A. Douse, K. T. Foley, Receptor interactions in modulating ventilatory activity. Am. J. Phys. Regul. Integr. Comp. Phys. 259, R911–R920 (1990). 10.1152/ajpregu.1990.259.5.R911 2240275

[62] R. J. Wilson, J. E. Remmers, J. F. Paton, Brain stem Po_2_ and pH of the working heart-brain stem preparation during vascular perfusion with aqueous medium. Am. J. Physiol. Regul. Integr. Comp. Physiol. 281, R528–R538 (2001). 11448857 10.1152/ajpregu.2001.281.2.R528

[63] T. A. Day, R. J. A. Wilson, A negative interaction between brainstem and peripheral respiratory chemoreceptors modulates peripheral chemoreflex magnitude. J. Physiol. 587, 883–896 (2009). 19103684 10.1113/jphysiol.2008.160689 PMC2669977

[64] E. S. Furfine, M. F. Harmon, J. E. Paith, E. P. Garvey, Selective inhibition of constitutive nitric oxide synthase by L-NG-nitroarginine. Biochemistry 32, 8512–8517 (1993). 7689333 10.1021/bi00084a017

[65] B. R. Babu, C. Frey, O. W. Griffith, l-arginine binding to nitric-oxide synthase. The role of H-bonds to the nonreactive guanidinium nitrogens. J. Biol. Chem. 274, 25218–25226 (1999). 10464242 10.1074/jbc.274.36.25218

[66] A. O. Caggiano, R. P. Kraig, Neuronal nitric oxide synthase expression is induced in neocortical astrocytes after spreading depression. J. Cereb. Blood Flow Metab. 18, 75–87 (1998). 9428308 10.1097/00004647-199801000-00008 PMC2698993

[67] J. Stefanska, R. Pawliczak, Apocynin: Molecular aptitudes. Mediators Inflamm. 2008, 1–10 (2008). 10.1155/2008/106507 PMC2593395 19096513

[68] H. L. Liang, G. Hilton, J. Mortensen, K. Regner, C. P. Johnson, V. Nilakantan, MnTMPyP, a cell-permeant SOD mimetic, reduces oxidative stress and apoptosis following renal ischemia-reperfusion. Am. J. Physiol. Renal Physiol. 296, F266–F276 (2009). 19091787 10.1152/ajprenal.90533.2008 PMC2643863

[69] C. M. Hecquet, A. B. Malik, Role of H_2_O_2_-activated TRPM2 calcium channel in oxidant-induced endothelial injury. Thromb. Haemost. 101, 619–625 (2009). 19350103 PMC3699330

[70] Y. Sawada, H. Hosokawa, K. Matsumura, S. Kobayashi, Activation of transient receptor potential ankyrin 1 by hydrogen peroxide. Eur. J. Neurosci. 27, 1131–1142 (2008). 18364033 10.1111/j.1460-9568.2008.06093.x

[71] M. Petrus, A. M. Peier, M. Bandell, S. W. Hwang, T. Huynh, N. Olney, T. Jegla, A. Patapoutian, A role of TRPA1 in mechanical hyperalgesia is revealed by pharmacological inhibition. Mol. Pain 3, 40 (2007). 18086313 10.1186/1744-8069-3-40 PMC2222610

[72] R. Gupta, S. Saito, Y. Mori, S. G. Itoh, H. Okumura, M. Tominaga, Structural basis of TRPA1 inhibition by HC-030031 utilizing species-specific differences. Sci. Rep. 6, 37460 (2016). 27874100 10.1038/srep37460 PMC5118716

[73] T. Grand, M. Demion, C. Norez, Y. Mettey, P. Launay, F. Becq, P. Bois, R. Guinamard, 9-Phenanthrol inhibits human TRPM4 but not TRPM5 cationic channels. Br. J. Pharmacol. 153, 1697–1705 (2008). 18297105 10.1038/bjp.2008.38 PMC2438271

[74] K. Togashi, H. Inada, M. Tominaga, Inhibition of the transient receptor potential cation channel TRPM2 by 2-aminoethoxydiphenyl borate (2-APB). Br. J. Pharmacol. 153, 1324–1330 (2008). 18204483 10.1038/sj.bjp.0707675 PMC2275460

[75] H. Iwasaki, Y. Mori, Y. Hara, K. Uchida, H. Zhou, K. Mikoshiba, 2-Aminoethoxydiphenyl borate (2-APB) inhibits capacitative calcium entry independently of the function of inositol 1,4,5-trisphosphate receptors. Recept. Channels 7, 429–439 (2001). 11918346

[76] R. Guinamard, C. Simard, C. Del Negro, Flufenamic acid as an ion channel modulator. Pharmacol. Ther. 138, 272–284 (2013). 23356979 10.1016/j.pharmthera.2013.01.012 PMC4116821

[77] Y. Okada, T. Sasaki, Y. Oku, N. Takahashi, M. Seki, S. Ujita, K. F. Tanaka, N. Matsuki, Y. Ikegaya, Preinspiratory calcium rise in putative pre-Bötzinger complex astrocytes. J. Physiol. 590, 4933–4944 (2012). 22777672 10.1113/jphysiol.2012.231464 PMC3487046

[78] H. Onimaru, I. Yazawa, K. Takeda, I. Fukushi, Y. Okada, Calcium imaging analysis of cellular responses to hypercapnia and hypoxia in the NTS of newborn rat brainstem preparation. Front. Physiol. 12, 645904 (2021). 33841182 10.3389/fphys.2021.645904 PMC8027497

[79] R. Dallwig, J. W. Deitmer, Cell-type specific calcium responses in acute rat hippocampal slices. J. Neurosci. Methods 116, 77–87 (2002). 12007985 10.1016/s0165-0270(02)00030-4

[80] K. J. Cummings, R. J. Wilson, Time-dependent modulation of carotid body afferent activity during and after intermittent hypoxia. Am. J. Physiol. Regul. Integr. Comp. Physiol. 288, R1571–R1580 (2005). 15677524 10.1152/ajpregu.00788.2004

[81] A. Roy, S. Mandadi, M.-N. Fiamma, E. Rodikova, E. V. Ferguson, P. J. Whelan, R. J. A. Wilson, Anandamide modulates carotid sinus nerve afferent activity via TRPV1 receptors increasing responses to heat. J. Appl. Physiol. 112, 212–224 (2012). 21903882 10.1152/japplphysiol.01303.2010

[82] E. H. Vidruk, E. B. Olson, L. Ling, G. S. Mitchell, Responses of single-unit carotid body chemoreceptors in adult rats. J. Physiol. 531, 165–170 (2001). 11179400 10.1111/j.1469-7793.2001.0165j.x PMC2278456

[83] M. Tani, I. Yazawa, K. Ikeda, K. Kawakami, H. Onimaru, Long-lasting facilitation of respiratory rhythm by treatment with TRPA1 agonist, cinnamaldehyde. J. Neurophysiol. 114, 989–998 (2015). 26108952 10.1152/jn.00282.2015 PMC4725117

[84] J. M. Hinrichs, I. J. Llewellyn-Smith, Variability in the occurrence of nitric oxide synthase immunoreactivity in different populations of rat sympathetic preganglionic neurons. J. Comp. Neurol. 514, 492–506 (2009). 19350665 10.1002/cne.22015

[85] Y. Mori, N. Takahashi, T. Kurokawa, S. Kiyonaka, TRP channels in oxygen physiology: Distinctive functional properties and roles of TRPA1 in O_2_ sensing. Proc. Jpn. Acad. Ser. B Phys. Biol. Sci. 93, 464–482 (2017). 10.2183/pjab.93.028 PMC5713176 28769017

