# Supplementary material for: Novel oxygen sensing mechanism in the spinal cord involved in cardiorespiratory responses to hypoxia
Source: Sci Adv. 2022 Mar 25;8(12):eabm1444. doi: 10.1126/sciadv.abm1444 (PMC8956269; doi:10.1126/sciadv.abm1444)
Supplement: Supplementary file 1 — Figs. S1 to S6 Tables S1 to S4 [file sciadv.abm1444_sm.pdf]

Science Advances
AAAS

Supplementary Materials for

Novel oxygen sensing mechanism in the spinal cord involved in cardiorespiratory responses to hypoxia

Nicole O. Barioni, Fatemeh Derakhshan, Luana Tenorio Lopes, Hiroshi Onimaru, Arijit Roy, Fiona McDonald, Erika Scheibli, Mufaddal I. Baghdadwala, Negar Heidari, Manisha Bharadia, Keiko Ikeda, Itaru Yazawa, Yasumasa Okada, Michael B. Harris, Mathias Dutschmann, Richard J. A. Wilson*

*Corresponding author. Email: wilsonr@ucalgary.ca

Published 25 March 2022, *Sci. Adv.* **8**, eabm1444 (2022)
DOI: 10.1126/sciadv.abm1444

This PDF file includes:

Figs. S1 to S6
Tables S1 to S4

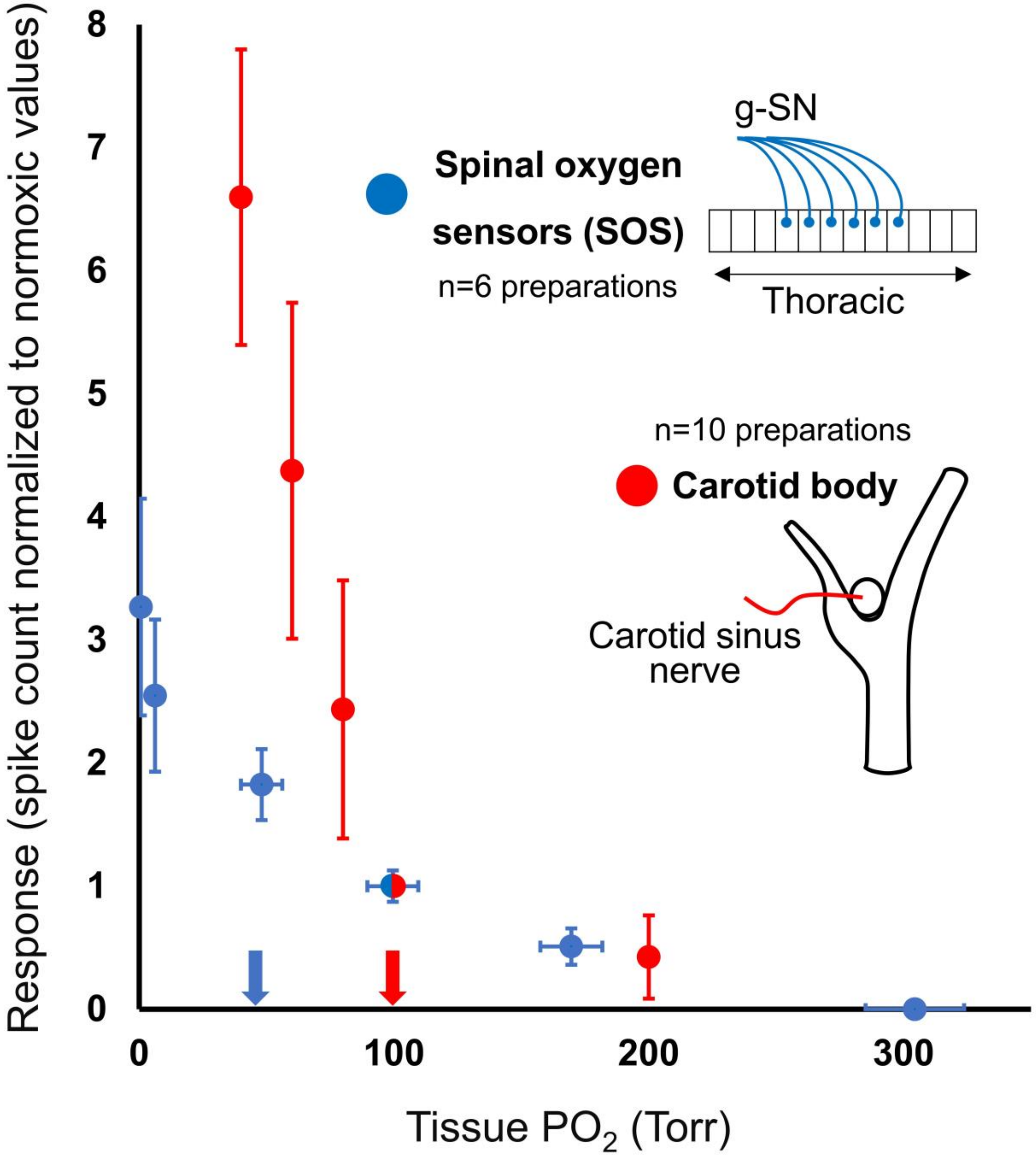

Supplementary Figure 1. Oxygen sensitivity of the SOS compared to the carotid body. SOS data from perfused *in situ* spinal cord preparation in **Figure 1E**, re-plotted normalized to activity at 100 Torr PO_2 to assist in comparison to carotid body data obtained from the *en bloc*, artificially perfused carotid body preparation (*80*). Blue and red arrows indicate *in vivo* normoxic tissue values of CNS and carotid bodies, respectively.

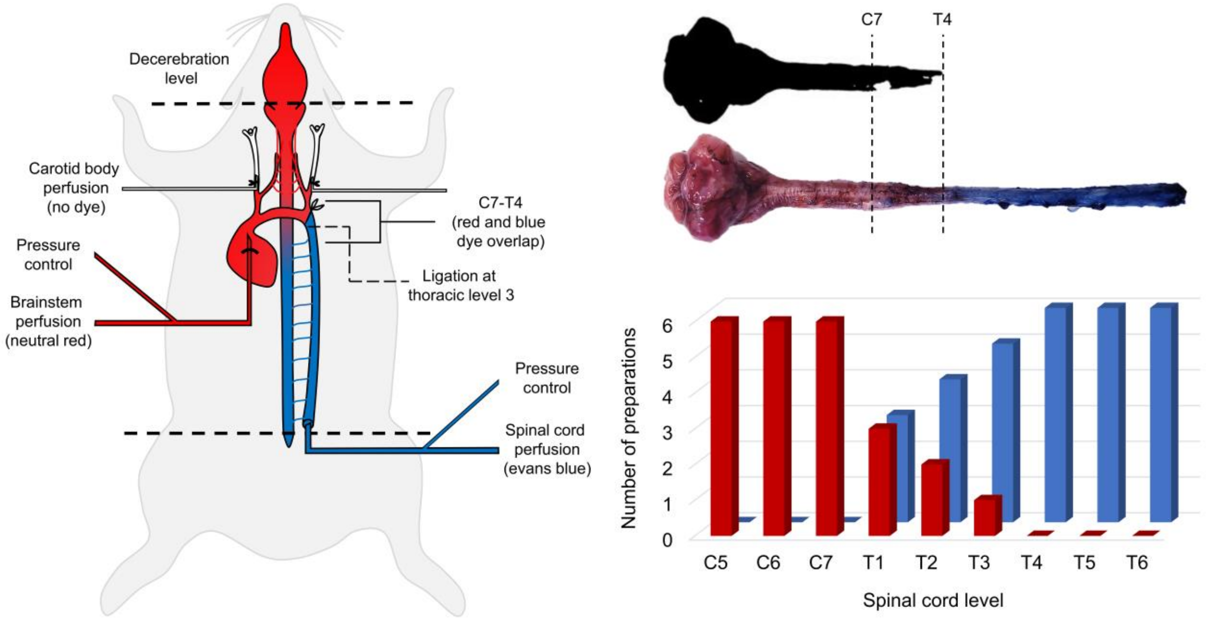

Supplementary Figure 2. Compartmentalization of artificial CNS perfusion in the *in situ* triple perfused preparation. Evans Blue (0.05%) was perfused in a retrograde direction through the descending aorta (thoracic spinal cord compartment) while Neutral Red (0.05%) was perfused through the heart (brainstem and cervical spinal cord compartment) as illustrated in *left panel*. Perfusion to the two compartments was separated by ligating the descending aorta at T3. The caudal extent of perfusion to the brainstem compartment was analyzed using automated software (*right panel; n=6*). *Red bars*, caudal extent of perfusion to the brainstem compartment*; Blue bars*, spinal segments only receiving perfusate delivered to the thoracic spinal cord compartment.

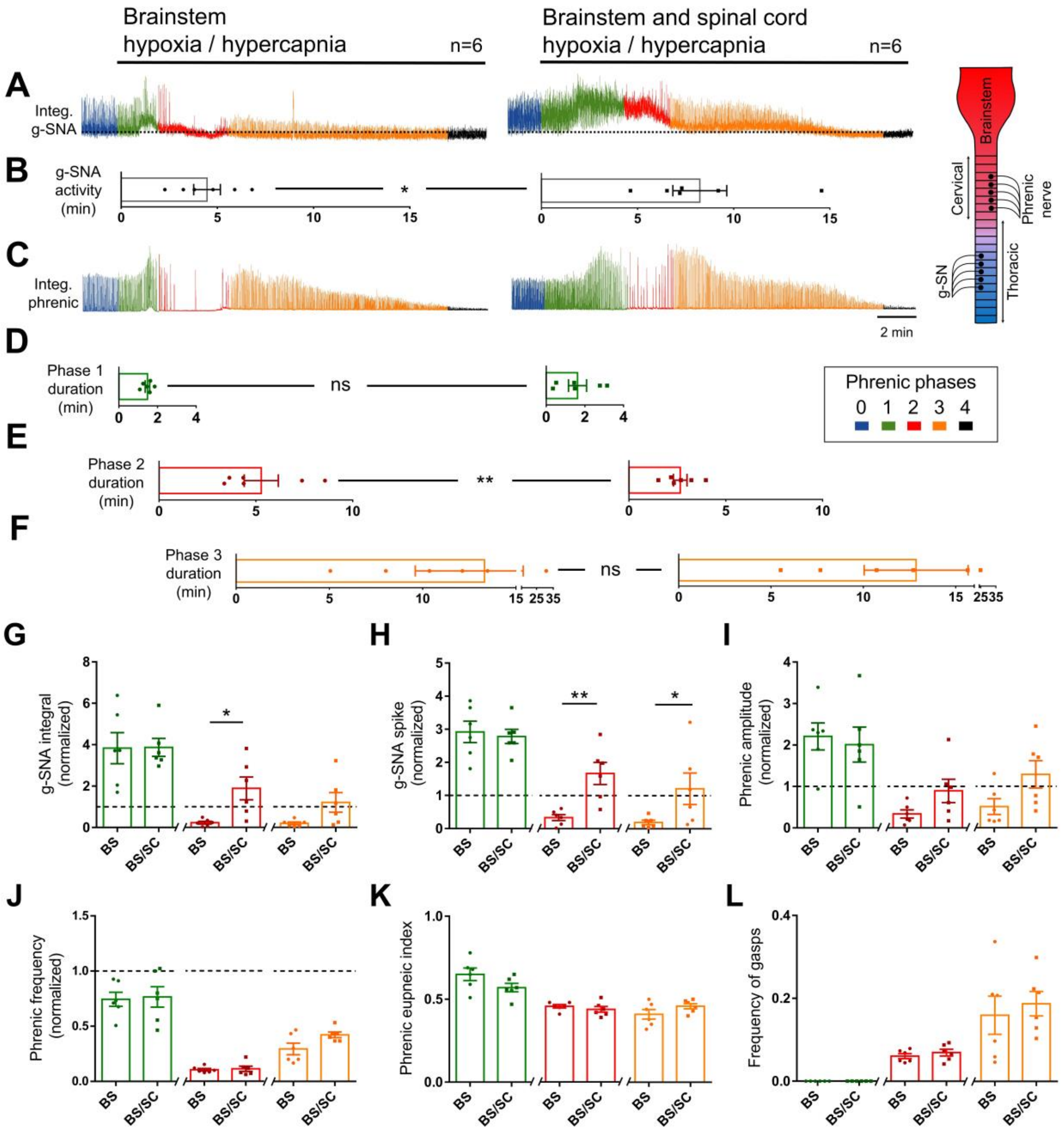

Brainstem
hypoxia / hypercapnia
n=6
Brainstem and spinal cord
hypoxia / hypercapnia
n=6
A
Integ.
g-SNA
B
g-SNA
activity
(min)
*
C
Integ.
phrenic
2 min
D
Phase 1
duration
(min)
ns
E
Phase 2
duration
(min)
**
F
Phase 3
duration
(min)
ns
Brainstem
Cervical
Phrenic
nerve
g-SN
Thoracic
Phrenic phases
0 1 2 3 4
G
g-SNA integral
(normalized)
H
g-SNA spike
(normalized)
I
Phrenic amplitude
(normalized)
J
Phrenic frequency
(normalized)
K
Phrenic eupneic index
L
Frequency of gasps
BS
BS/SC

Supplementary Figure 3: SOS promotes defensive sympatho-respiratory responses to asphyxia-like stimuli. Triple perfused *in situ* preparation with independent perfusion of carotid bodies, brainstem (+spinal levels C1-T3) and thoracic spinal cord (T4-T13) compartments. For verification of brainstem and thoracic spinal cord compartment independence, see **SFigure 2.** Carotid body perfusate was kept constant throughout (35 Torr PCO_2, 100 Torr PO_2). Brainstem alone or brainstem plus thoracic compartment were challenged with hypoxic-hypercapnic perfusate (PO_2 40 Torr / PCO_2 60 Torr). Respiratory responses divided into 5 phases based on phrenic nerve activity (*box*): **0**, baseline; **1**, hyperpnea; **2**, primary apnea and gasp; **3**, recovery phase; **4**, terminal apnea. (**A,B**) exposing the brainstem compartment alone (*left panel)* causes an transient increase in phrenic activity (Phase 1) followed by primary apnea and gasps (Phase 2). Compared to exposing the brainstem compartment alone, exposing brainstem and thoracic compartments (*right panel*), prolongs greater splanchnic nerve activity (g-SNA; time to decay to 50% below baseline: unpaired t test with Welch's correction P=0.045); (**C-F**) shortens the apnea/gasp phase (Phase 2), promoting faster onset of recovery (Phase 3; Mann-Whitney test, P=0.0087); (**G-H**) increases g-SNA during Phases 2 and 3 (unpaired t test with Welch's correction, integral Phase 2: P=0.029, spike Phase 2: P=0.009, spike Phase 3 P=0.042); (**I-L**), has no effect on phrenic burst amplitude, shape or frequency beyond that of exposing the brainstem alone. Integ. g-SNA/phrenic: integrated and rectified greater splanchnic/phrenic nerve activity; amplitude and/or area under curve indicates activity level. Bars show mean ± SEM. *P<0.05, **P<0.01.

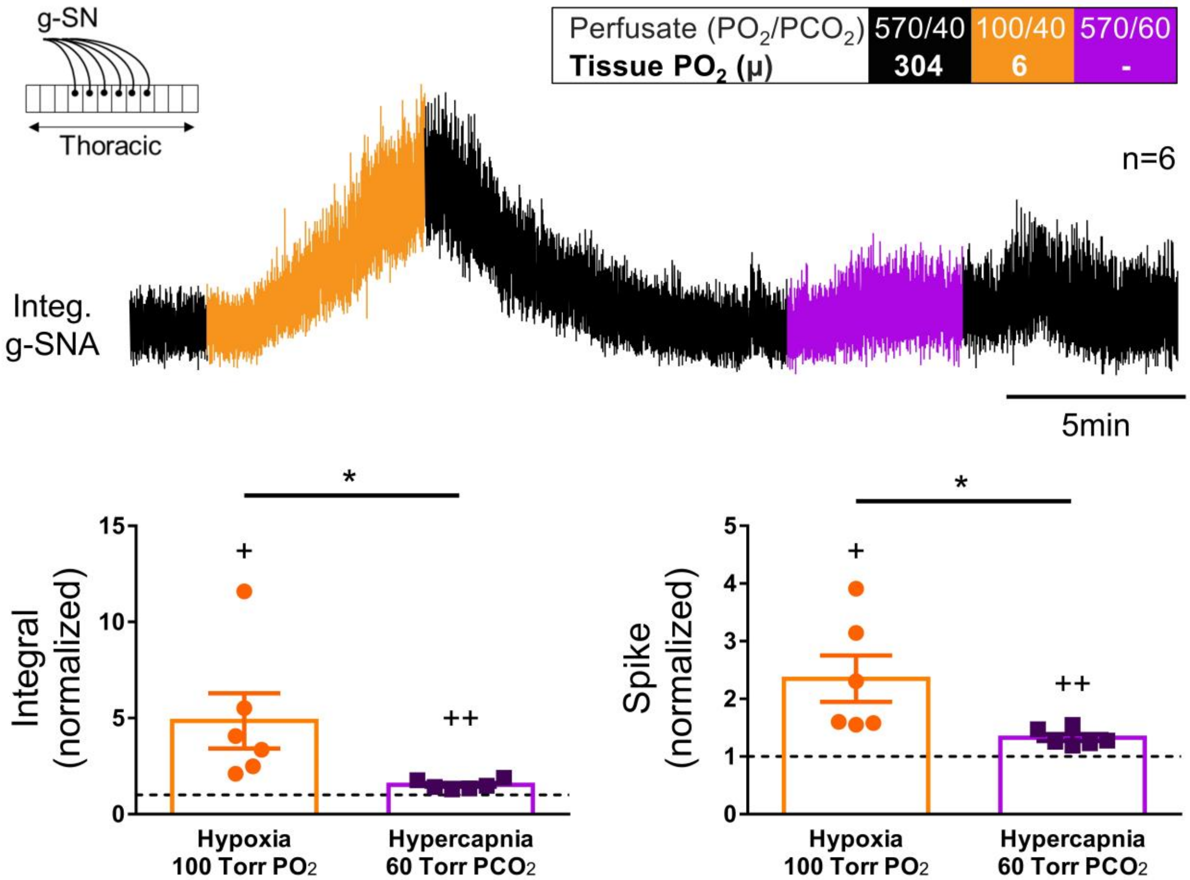

g-SN
Thoracic
Perfusate (PO2/PCO2) 570/40 100/40 570/60
Tissue PO2 (μ) 304 6 -
n=6
Integ. g-SNA
5min
Integral (normalized)
Spike (normalized)
Hypoxia 100 Torr PO2
Hypercapnia 60 Torr PCO2

Supplementary Figure 4: The SOS are mildly CO_2 sensitive. *Top panel,* recording of greater splanchnic nerve activity (g-SNA) in response to 5-min bouts of hypoxia (100 Torr PO_2 / 40 Torr PCO_2; average tissue: 6.2 ± 10 Torr PO_2) or hypercapnia (570 Torr PO_2 / 60 Torr PCO_2) in a single perfused thoracic spinal cord *in situ* preparation (schematic in *top left corner*). Perfusion between bouts was returned to 570 Torr PO_2 / 40 Torr PCO_2 for 5 min. *Bottom panel,* group data (n=6). Reducing perfusate PO_2 or increasing perfusate PCO_2 increases g-SNA (one-sample t test - difference from baseline: hypoxia integral, P=0.044 and spike, P=0.02; hypercapnia integral, P=0.0029 and spike, P=0.0026). However, the hypoxic response is significantly larger than the hypercapnic response (ratio paired t test - integral, P=0.012, and spike, P=0.027). Integ. g-SNA: integrated and rectified greater splanchnic nerve activity; amplitude and/or area under curve indicates activity level. *Box*: color-coding illustrates perfusate PO_2 and PCO_2 in Torr.

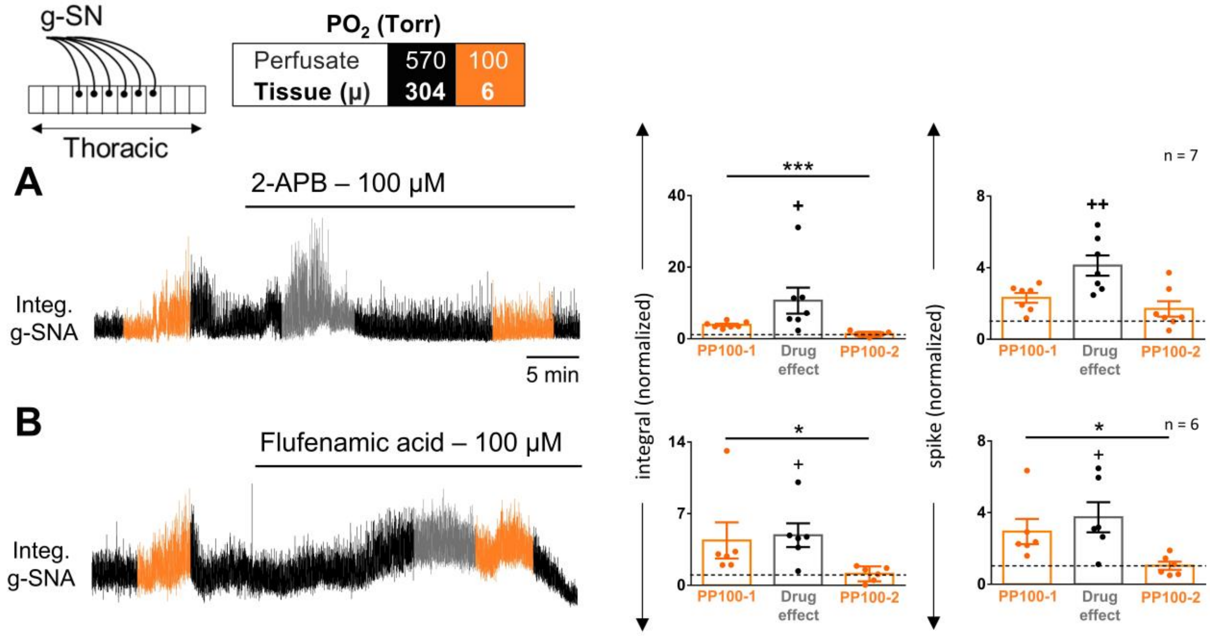

Supplementary Figure 5. Effect of broadband receptor antagonists in the SOS O_2 sensing mechanism. *Box*: color-coding illustrates perfusate PO_2 (PP) in Torr. *Left*, g-SNA responses to hypoxia in rat *in situ* thoracic spinal cord preparations (*see schematic*). The g-SN responses and drug effects were measured during the 1 min interval with the greatest activity, illustrated in orange (perfusate and tissue PO_2 100 and ~6 Torr, respectively) and gray (drug). *Right*, group data. (**A**) 2-APB; broadband TRP and $IP3_R$ blocker and (**B**) Flufenamic Acid, broadband TRP channel blocker. Dots show data from individual preparations; values exceeding dashed lines are greater than baseline. Bars show mean ± SEM. Comparison between hypoxic responses before and with drug: *P<0.05, ***P≤0.001 Baseline effect of drug: $^{+}$P<0.05, $^{++}$P<0.01. Integ. g-SNA: integrated and rectified greater splanchnic nerve activity; amplitude and/or area under curve indicates activity level. Time scale in **A** applies to all traces unless otherwise shown. See **STable 2** for detailed statistics.

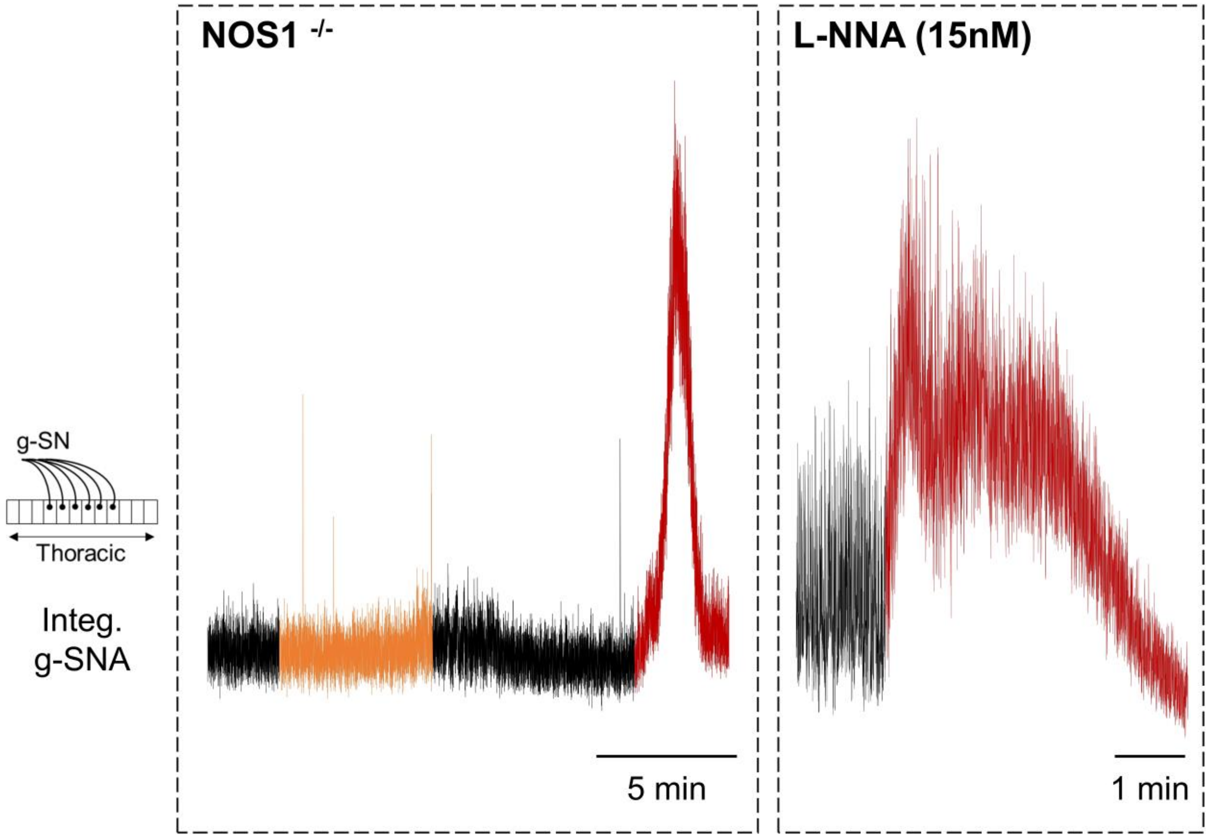

Supplementary Figure 6. Ischemia persists in the absence of NOS1 activity. g-SNA responses of *in situ* thoracic spinal cord preparations (*see schematic*) to hypoxia (orange, perfusate PO_2 100 Torr) and/or ischemia (red, stop flow) in NOS1 KO mouse (left) and rat treated with L-NNA (15 nM, right). Interrupting perfusate supply to preparations when NOS1 production of NO is curtailed leads to increase in splanchnic nerve activity.

Supplementary Table 1 - Patch Clamp data of control only (rats)

Experiment	Normality test	Statistical tests used	P value
**Membrane potential**	All passed	Repeat measures 1-way ANOVA with Dunnett multiple comparisons test of baseline vs hypoxia, and baseline vs washout	Baseline *vs* hypoxia = 0.022; Baseline *vs* washout = 0.26
**Input resistance**	All passed	Repeat measures 1-way ANOVA with Dunnett multiple comparisons test of baseline vs hypoxia, and baseline vs washout	Baseline *vs* hypoxia = 0.59; Baseline *vs* washout = 0.28

Supplementary Table 2 – pharmacology g-SNA recording data (rats)

Experiment	Aspect	Normality test	Statistical tests used	P value
**Gliotransmitter and synapse cocktail (n=6)**	Integral	PP100-2 did not pass	Friedman test with Dunn's multiple comparisons test between all Hx challenges; drug effect and washout - one sample t test (against 1)	PP100-1 *vs* PP100-2 *vs* PP100-3 > 0.99; drug effect = 0.15; washout = 0.17
	Spike	PP100-2 did not pass	Friedman test with Dunn's multiple comparisons test between all Hx challenges; drug effect and washout - one sample t test (against 1)	PP100-1 *vs* PP100-2 *vs* PP100-3 > 0.99; drug effect = 0.055; washout = 0.13
**Fluorocitric acid (n=6)**	Integral	Drug effect did not pass	PP100-1 vs PP100-2 - paired t test; drug effect - Wilcoxon test (against 1)	PP100-1 vs PP100-2 = 0.35; drug effect = 0.031
	Spike	Drug effect did not pass	PP100-1 vs PP100-2 - paired t test; drug effect - Wilcoxon test (against 1)	PP100-1 *vs* PP100-2 = 0.15; drug effect = 0.031
**Time control (n=5)**	Integral	PP100-2 did not pass	PP100-1 vs PP100-22 - Wilcoxon test; drug effect - one sample t test (against 1)	PP100-1 *vs* PP100-2 = 0.44; drug effect = 0.066

	Spike	All passed	PP100-1 vs PP100-2 - paired t test; drug effect - one sample t test (against 1)	PP100-1 *vs* PP100-2 = 0.95; drug effect = 0.47
**Nω-nitro-l-arginine (L-NNA; n=6)**	Integral	PP100-1 and drug effect did not pass	PP100-1 vs PP100-2 - Wilcoxon test; drug effect - Wilcoxon test (against 1)	PP100-1 *vs* PP100-2 = 0.031; drug effect = 1
	Spike	PP100-1 did not pass	PP100-1 vs PP100-2 - Wilcoxon test; drug effect - one sample t test (against 1)	PP100-1 *vs* PP100-2 = 0.031; drug effect = 0.88
**L-arginine (n=6)**	Integral	All passed	PP100-1 vs PP100-2 - ratio paired t test; drug effect - one sample t test (against 1)	PP100-1 *vs* PP100-2 = 0.0091; drug effect = 0.068
	Spike	Drug effect did not pass	PP100-1 vs PP100-2 - ratio paired t test; drug effect - Wilcoxon test (against 1)	PP100-1 *vs* PP100-2 = 0.0011; drug effect = 0.031
**Sodium Nitroprusside (SNP;n=5)**	Integral	All passed	PP100-1 vs PP100-2 - ratio paired t test; drug effect - one sample t test (against 1)	PP100-1 *vs* PP100-2 = 0.61; drug effect = 0.028
	Spike	All passed	PP100-1 vs PP100-2 - ratio paired t test; drug effect - one sample t test (against 1)	PP100-1 *vs* PP100-2 = 0.59; drug effect = 0.021
**Apocynin (n=6)**	Integral	All passed	PP100-1 vs PP100-2 - ratio paired t test; drug effect - one sample t test (against 1)	PP100-1 *vs* PP100-2 = 0.014; drug effect = 0.095
	Spike	All passed	PP100-1 vs PP100-2 - ratio paired t test; drug effect - one sample t test (against 1)	PP100-1 *vs* PP100-2 = 0.0074; drug effect = 0.41
**Gadolinium (n=7)**	Integral	PP100-1 and drug effect did not pass	PP100-1 vs PP100-2 - Wilcoxon test; drug effect - Wilcoxon test (against 1)	PP100-1 *vs* PP100-2 = 0.58; drug effect = 0.031
	Spike	PP100-1 and drug effect did not pass	PP100-1 vs PP100-2 - Wilcoxon test; drug effect - Wilcoxon test (against 1)	PP100-1 *vs* PP100-2 = 0.47; drug effect = 0.031
**Mn(III)tetrakis(1-methyl-4-pyridyl)porphyrin (MnTMPyP; n=6)**	Integral	All passed	PP100-1 vs PP100-2 - ratio paired t test; drug effect - one sample t test (against 1)	PP100-1 *vs* PP100-2 = 0.66; drug effect = 0.038

	Spike	All passed	PP100-1 vs PP100-2 - paired t test; drug effect - one sample t test (against 1)	PP100-1 *vs* PP100-2 = 0.49; drug effect = 0.021
**H_2O_2 (n=6)**	Integral	All passed	PP100-1 vs PP100-2 - ratio paired t test; drug effect - one sample t test (against 1)	PP100-1 *vs* PP100-2 = 0.037; drug effect = 0.036
	Spike	All passed	PP100-1 vs PP100-2 - ratio paired t test; drug effect - one sample t test (against 1)	PP100-1 *vs* PP100-2 = 0.038; drug effect = 0.027
**2-Aminoethyl diphenylborinate (2-APB; n=7)**	Integral	Drug effect did not pass	PP100-1 vs PP100-2 - paired t test; drug effect - Wilcoxon test (against 1)	PP100-1 *vs* PP100-2 = 0.0006; drug effect = 0.016
	Spike	PP100-2 did not pass	PP100-1 vs PP100-2 - Wilcoxon test; drug effect - one sample t test (against 1)	PP100-1 *vs* PP100-2 = 0.3; drug effect = 0.0015
**Flufenamic acid (n=6)**	Integral	PP100-1 and drug effect did not pass	PP100-1 vs PP100-2 - Wilcoxon test; drug effect - Wilcoxon test (against 1)	PP100-1 *vs* PP100-2 = 0.031; drug effect = 0.031
	Spike	PP100-1 did not pass	PP100-1 vs PP100-2 - Wilcoxon test; drug effect - one sample t test (against 1)	PP100-1 *vs* PP100-2 = 0.031; drug effect = 0.022
**4-(4-Chlorophenyl)-3-methylbut-3-en-2-oxime (AP-18; n=6)**	Integral	All passed	repeat measures 2-way ANOVA between all Hx challenges; drug effect - one sample t test (against 1)	PP300-1 *vs* *PP*300-2 = 0.9; PP100-1 *vs* *PP*100-2 = 0.0081; drug effect = 0.058
	Spike	PP300-2 did not pass	repeat measures 2-way ANOVA between all Hx challenges; drug effect - one sample t test (against 1)	PP300-1 *vs* PP300-2 = 0.93; PP100-1 *vs* *PP*100-2 = 0.0003; drug effect = 0.043
**1,2,3,6-Tetrahydro-1,3-dimethyl-N-[4-(1-methylethyl)phenyl]-2,6-dioxo-7H-purine-7-acetamide (HC030031; n=6)**	Integral	PP100-1 and drug effect did not pass	repeat measures 2-way ANOVA between all Hx challenges; drug effect - Wilcoxon test (against 1)	PP300-1 *vs* PP300-2 = 0.48; PP100-1 *vs* *PP*100-2 = 0.02; drug effect = 0.031
	Spike	All passed	repeat measures 2-way ANOVA between all Hx challenges; drug effect -	PP300-1 *vs* PP300-2 = 0.8; PP100-1 *vs*

			one sample t test (against 1)	*PP*100-2 = 0.036; drug effect = 0.17
**9-phenanthrol (n=6)**	Integral	All passed	PP100-1 vs PP100-2 - ratio paired t test; drug effect - one sample t test (against 1)	PP100-1 *vs* PP100-2 = 0.047; drug effect = 0.17
	Spike	All passed	PP100-1 vs PP100-2 - ratio paired t test; drug effect - one sample t test (against 1)	PP100-1 *vs* PP100-2 = 0.12; drug effect = 0.065
**Hypercapnia (n=6)**	Integral	All passed	PP100 vs $PPCO_260$ - ratio paired t test; one sample t test (against 1)	PP100 *vs* $PPCO_260$ = 0.012; PP100 = 0.043; $PPCO_260$ = 0.003
	Spike	All passed	PP100 vs $PPCO_260$ - ratio paired t test; one sample t test (against 1)	PP100 *vs* $PPCO_260$ = 0.027; Hx100 = 0.02; $PPCO_260$ = 0.0026
**Hexamethonium (n=6)**	Spike	PP100 and PP60 did not pass	Friedman test with Dunn's multiple comparisons test of all Hx challenges vs baseline	PP400 = 0.35; PP300 = 0.086; PP200 = 0.012; PP100 = 0.006; PP60 = 0.0007

PP100-1 and PP100-2: response to perfusate PO_2 of 100 (tissue PO_2 ~6) Torr before and with drug, respectively; PP300-1 and PP300-2: response to perfusate PO_2 of 300 (tissue PO_2 of ~100) Torr before and with drug, respectively. PP400, PP300, PP200, PP100 and PP60 are responses to perfusate PO_2 of 400, 300, 200, 100 and 60 Torr respectively (See **Figure 1** for tissue PO_2 reference); $PPCO_260$: perfusate PCO_2 of 60 Torr.

Supplementary Table 3 - g-SNA recording data (mice)

Experiment	Aspect	Normality test	Statistical tests used	P value
**NOS1 KO vs WT (n=6 each)**	Integral	KO PP100 did not pass	Repeat measures 2-way ANOVA between hx challenges	WT PP300 *vs* KO PP300 = 0.041; WT PP100 *vs* KO PP100 < 0.0001
	Spike	WT PP300 and PP100, and KO PP100 did not pass	Repeat measures 2-way ANOVA between hx challenges	WT PP300 *vs* KO PP300 = 0.78; WT PP100 *vs* KO PP100 = 0.0016

**NOX2 KO vs WT (n=6 each)**	Integral	KO PP300 did not pass	Repeat measures 2-way ANOVA between hx challenges	WT PP300 *vs* KO PP300 = 0.16; WT PP100 *vs* KO PP100 = 0.0002
	Spike	WT PP300 and PP100, and KO PP300 did not pass	Repeat measures 2-way ANOVA between hx challenges	WT PP300 *vs* KO PP300 = 0.48; WT PP100 *vs* KO PP100 = 0.0004
**TRPA1 KO vs WT (n=6 each)**	Integral	KO PP100 did not pass	Repeat measures 2-way ANOVA between hx challenges	WT PP300 *vs* KO PP300 = 0.87; WT PP100 *vs* KO PP100 = 0.32
	Spike	WT PP300 and PP100, and KO PP300 did not pass	Repeat measures 2-way ANOVA between hx challenges	WT PP300 *vs* KO PP300 = 0.95; WT PP100 *vs* KO PP100 = 0.38

PP300 and PP100: response to perfusate PO_2 of 300 and 100 (tissue PO_2 of ~100 and ~6) Torr, respectively; WT and KO: wild type and knockout, respectively.

Supplementary Table 4 – pharmacology Patch Clamp data (rats)

Experiment	Aspect	Normality test	Statistical tests used	P value
**Membrane potential**	Control (n=10) +HC030031 (n=8) + 9-PNT (n=7)	Control did not pass	Kruskal-Wallis test with Dunn's multiple comparisons between control, HC030031 and 9-PNT	Control *vs* HC030031 = 0.0029; Control *vs* 9-PNT = 0.0326;
**Input resistance**	Control (n=10) +HC030031 (n=8) +9-PNT (n=7)	All passed	One sample t test against 1	Control = 0.13; HC030031 = 0.0029; 9-PNT = 0.3;
**Flickering**	Control (n=10) +HC030031 (n=8) +9-PNT (n=7)	All passed	One sample t test against 1	Control = 0.004; HC030031 = 0.19; 9-PNT = 0.25;

9-PNT: 9-phenanthrol
